# Supplementary material for: FOXQ1 recruits the MLL complex to activate transcription of EMT and promote breast cancer metastasis
Source: Nat Commun. 2022 Nov 1;13:6548. doi: 10.1038/s41467-022-34239-z (PMC9626503; doi:10.1038/s41467-022-34239-z)
Supplement: Supplementary file 1 — Supplementary Information [file 41467_2022_34239_MOESM1_ESM.pdf]

Supplementary Information for:

FOXQ1 Recruits the MLL Complex to Activate Transcription of EMT and Promote Breast Cancer Metastasis

Allison V. Mitchell, Ling Wu, C. James Block, Mu Zhang, Justin Hackett, Douglas B. Craig, Wei Chen, Yongzhong Zhao, Bin Zhang, Yongjun Dang, Xiaohong Zhang, Shengping Zhang, Chuangui Wang, Heather Gibson, Lori A. Pile, Benjamin Kidder, Larry Matherly, Zhe Yang, Yali Dou, Guojun Wu#

#Corresponding Author: Guojun Wu, Ph.D. wugu@karmanos.org

The PDF include:

Supplementary Figures:

Supplementary Figure 1. Binding of the MLL core complex is biochemically and functionally specific to FOXQ1.

Supplementary Figure 2. FOXQ1 and RbBP5 co-localize within EMT promoters.

Supplementary Figure 3. RbBP5 binding within EMT promoters is dependent upon FOXQ1.

Supplementary Figure 4. Extended validation of the FOXQ1 Forkhead box binding to RbBP5.

Supplementary Figure 5. Disruption of RbBP5 recruitment prevents activation of the FOXQ1 EMT program.

Supplementary Figure 6. Targeting the MLL core complex decreases the FOXQ1-driven oncogenic phenotype *in vitro.*

Supplementary Figure 7. Expression of FOXQ1 RbBP5-defective mutants results in a decrease in FOXQ1-driven oncogenic potential *in vitro*.

Supplementary Figure 8. Disruption of the FOXQ1-RbBP5 interaction results in an inhibition of tumor progression *in vivo*.

Supplementary Tables:

Supplementary Table 1. Sequence of gene-specific shRNAs.

Supplementary Table 2. PCR primers, Cloning primers and mutagenesis primers

Supplementary Table 3. Antibodies

Supplementary Figure 1

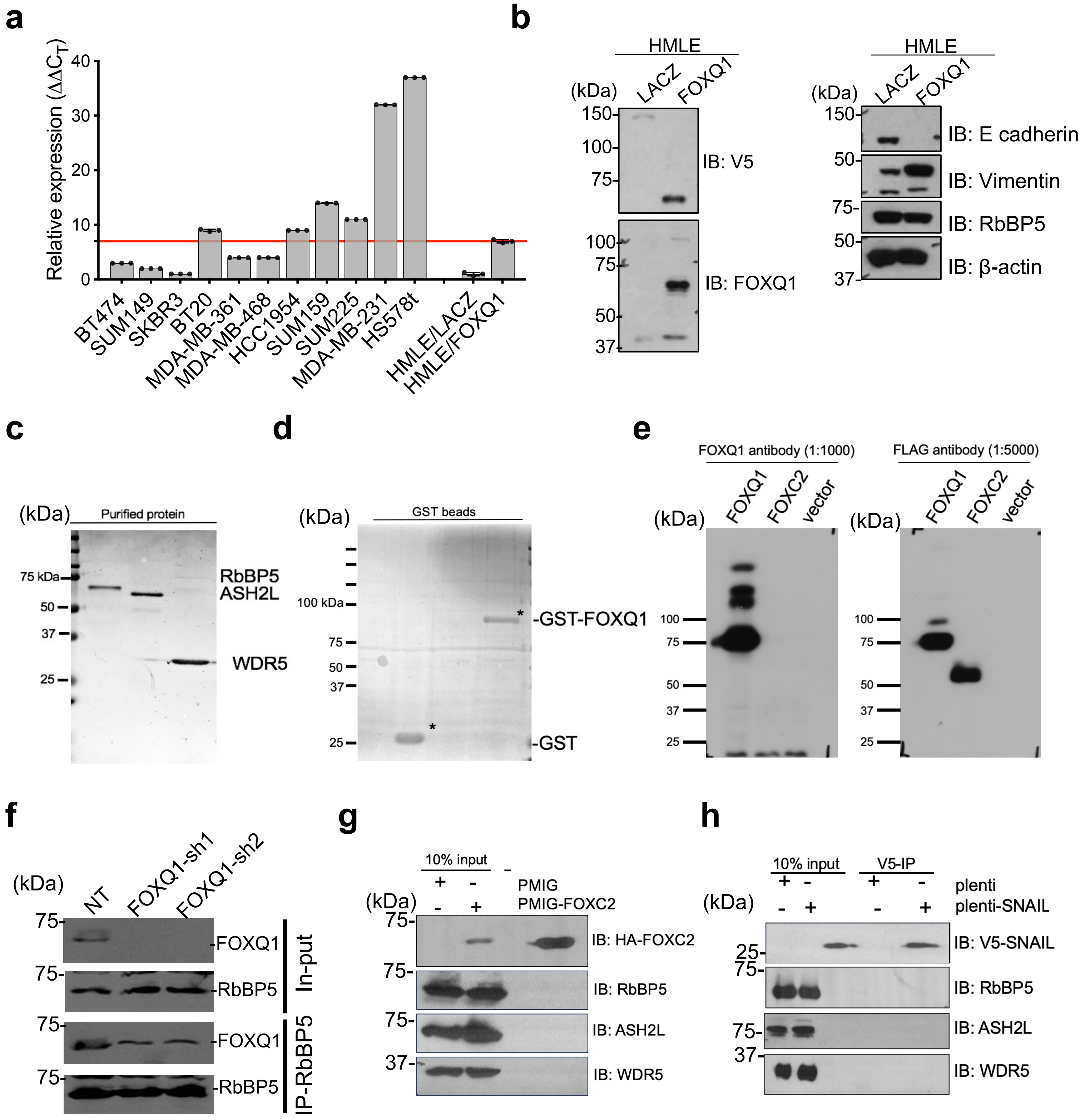

Supplementary Figure 1. Binding of the MLL core complex is biochemically and functionally specific to FOXQ1. (a) Quantification of FOXQ1 mRNA expression in a panel of breast cancer model cell lines by qRT-PCR. Values were normalized to β-actin relative to HMLE/LacZ control cells. Bar marks mean +/- SD. Data points mark values for independent replicates (n=3). The red line marks the relative FOXQ1 expression in the HMLE/FOXQ1 cells, which shows that the FOXQ1 ectopic expression is comparable to the endogenous FOXQ1 expression observed in TNBC cell lines. Source data are provided in Source Data file 1. **(b)** Human mammary luminal epithelial cells (HMLE) with stable lentiviral expression of V5-tagged FOXQ1 or LacZ vector control were analyzed by western blot and probed with indicated antibodies to determine relative protein expression. **(c)** Image of SDS-PAGE results of recombinant, untagged MLL core complex proteins (RbBP5, ASH2L, WDR5) expressed in *E. coli* and purified for use as prey for GST pull down. **(d)** Recombinant GST-FOXQ1, or GST control resin was examined by SDS-PAGE and subsequently incubated at equimolar ratio with MLL core complex proteins for GST pull down experiments. **(e)** Validation of FOXQ1 antibody. HEK293 cells were transfected with FLAG-FOXQ1, FLAG-FOXC2 or vector control. Membrane was probed with anti-FOXQ1 antibody from Rabbit serum. FLAG probed membrane serves as negative control**. (f)** MDA-MB231 cells with nontarget (NT) control and FOXQ1 knockdown. 10% in-put (two top panels) and protein lyses precipitated with RbBP5 antibody (two bottom panels) were probed with FOXQ1 antibody. **(g)** Co-IP in HEK293T cells with transient overexpression of HA-tagged FOXC2, or empty vector control (PMIG-HA). HA-IPs were probed with antibodies against endogenous MLL core complex proteins (RbBP5, ASH2L, WDR5). **(h)** Co-IP in HEK293T cells with transient overexpression of plenti-SNAIL1-V5. V5-IPs were probed with antibodies against endogenous MLL core complex proteins (RbBP5, ASH2L, WDR5). For panels 1b-h, representative images are shown (n=3).

Supplementary Figure 2

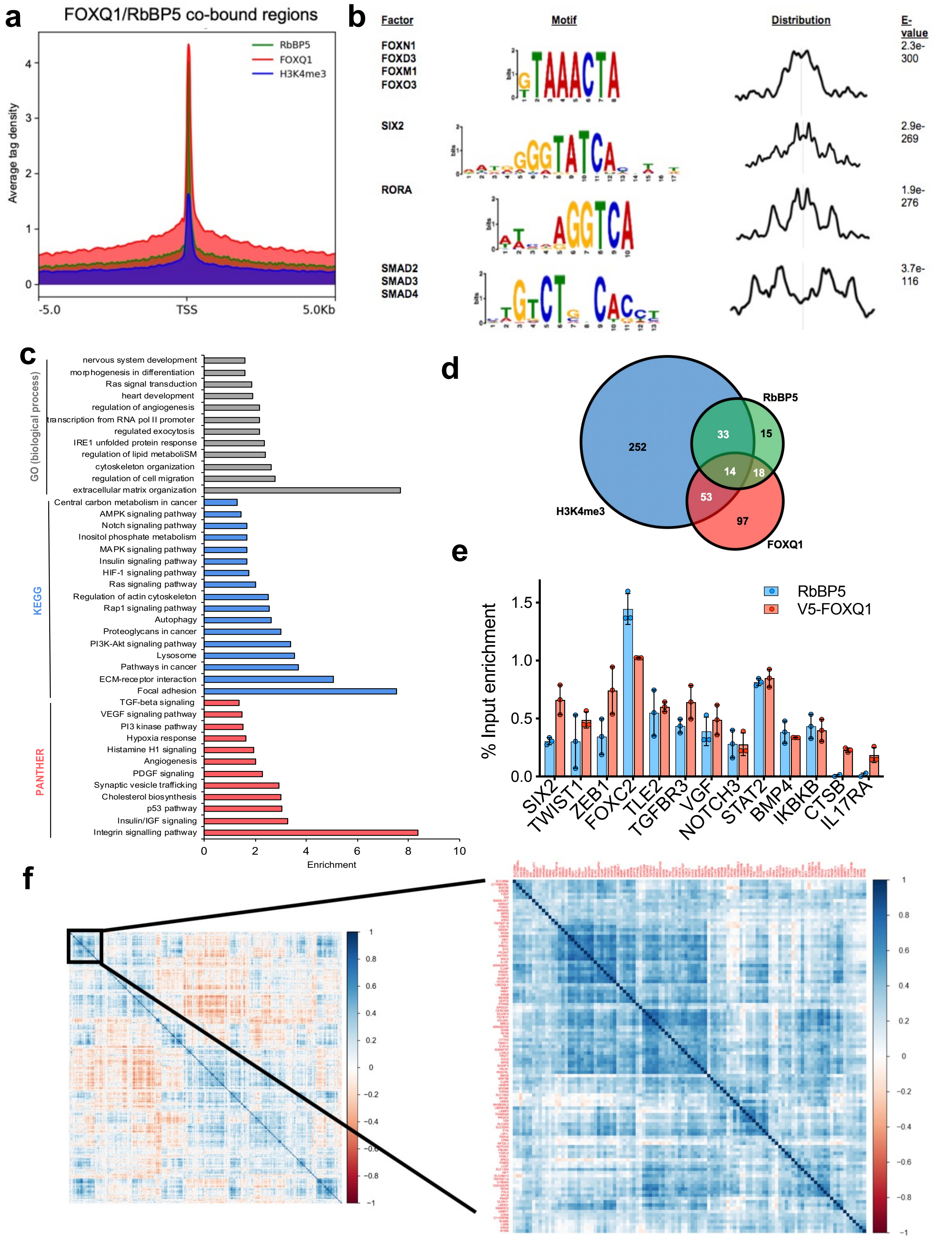

g

**KEGG**	**P-value**	**Adjusted p-value**
Insulin resistance	0.0003266	0.04376
Focal adhesion	0.0008423	0.05643

**ARCHS4 Kinase Coexpression**	**P-value**	**Adjusted p-value**
PDGFRB	7.726e-23	2.627e-20
DDR2	1.625e-21	2.763e-19
MYLK	3.233e-20	3.664e-18

h

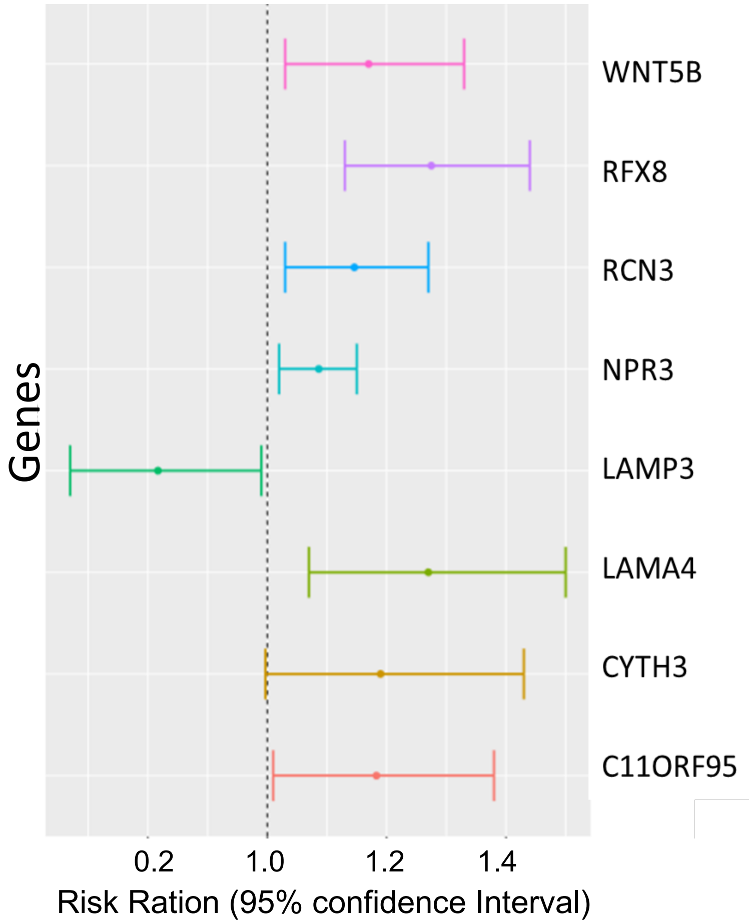

Supplementary Figure 2. FOXQ1 and RbBP5 co-localize within EMT promoters. **(a)** FOXQ1, RbBP5 and H3K4me3 ChIP-seq signal distribution within FOXQ1-RbBP5 overlapped chromatin regions. Results are plotted centered around TSS ± 5 kb. **(b)** TF binding motifs identified within the FOXQ1-RbBP5 co-bound regions (MEME). **(c)** Enrichment analysis of the 2,499 differentially upregulated genes identified in HMLE/FOXQ1 cells relative to HMLE/LacZ using Enrichr. Adjusted p-value was derived by comparison to annotated gene sets by two-sided Fisher's exact t-test with Benjamini-Hochberg correction. Graph displays the log-transformed adjusted p-value (Enrichment). (**d**) Venn diagram of the overlap of FOXQ1, RbBP5 and H3K4me3 levels within promoter regions of genes that are differentially downregulated in HMLE/FOXQ1 cells relative to HMLE/LacZ. **(e)** Validation of FOXQ1-RbBP5 co-binding within the promoter region of EMT-associated genes by ChIP-qPCR in HMLE/FOXQ1 cells. IL17RA and CTSB were selected as RbBP5-independent control genes. Percent input enrichment was obtained by comparing the ChIP-enriched signals to the input chromatin sample. Bar marks sample mean +/- SD. The values for each replicate shown (n=3). Source data are provided in Source Data 1. **(f)** Heatmap depicts the Spearman correlation coefficients for the FOXQ1-RbBP5 signature gene set (622 genes) (left). The top 109 correlated genes (Spearman R>0.2, boxed) within the FOXQ1-RbBP5 gene signature were replotted(right). (**g)** Enrichment analysis was performed on the 109 highly correlated FOXQ1/RbBP5-regulated using Enrichr. The gene set was compared to the indicated annotation datasets by two-sided Fisher's exact t-test with Benjamini-Hochberg correction. (**h)** Forest plot of the hazard ratios + 95% confidence interval for FOXQ1/RbBP5 regulated genes associated with overall survival in a multivariate Cox proportional hazards model. Several FOXQ1/RbBP5-regulated genes were associated with worse overall survival. One FOXQ1-RbBP5 associated gene, LAMP3, showed an opposite protective effect.

.

Supplementary Figure 3

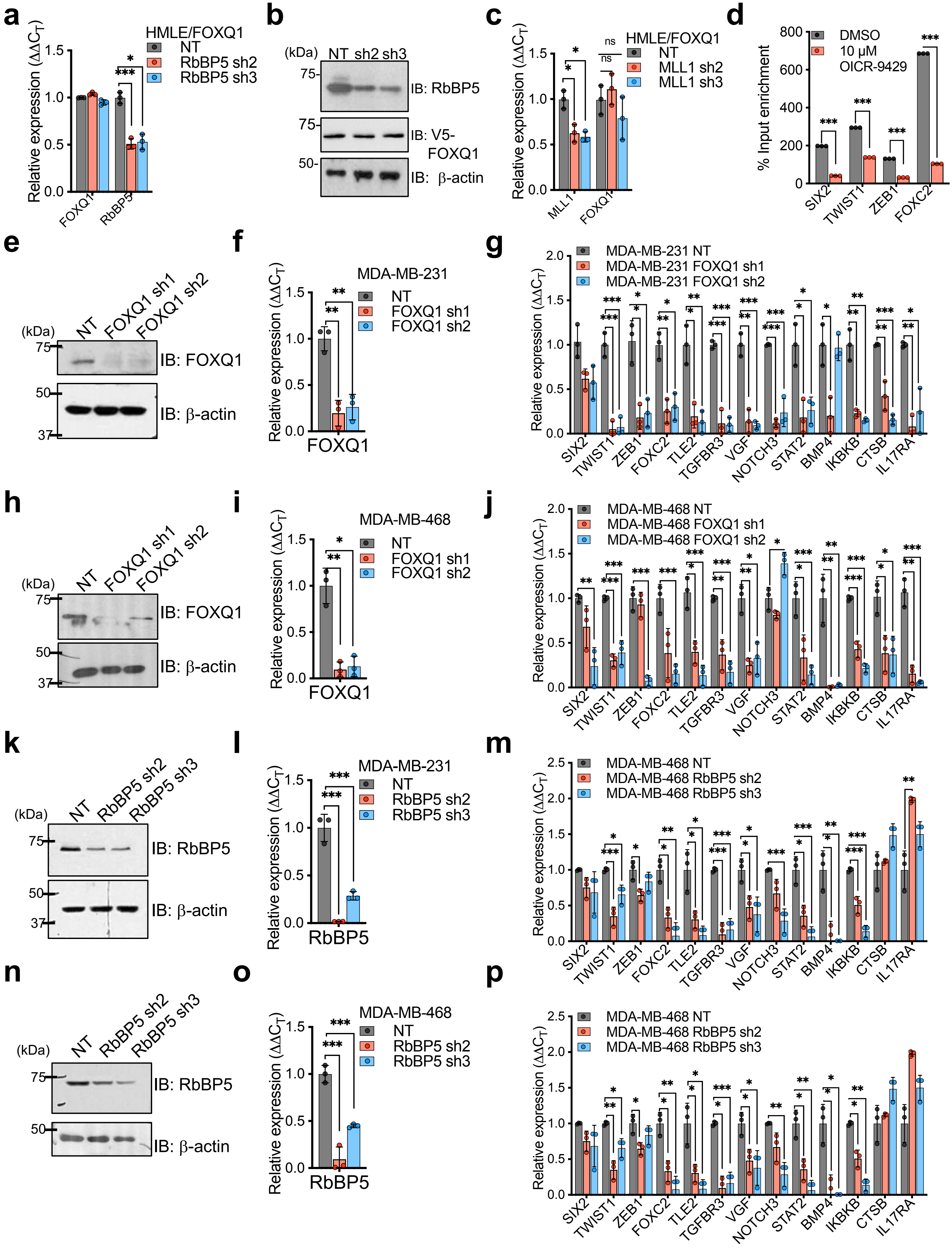

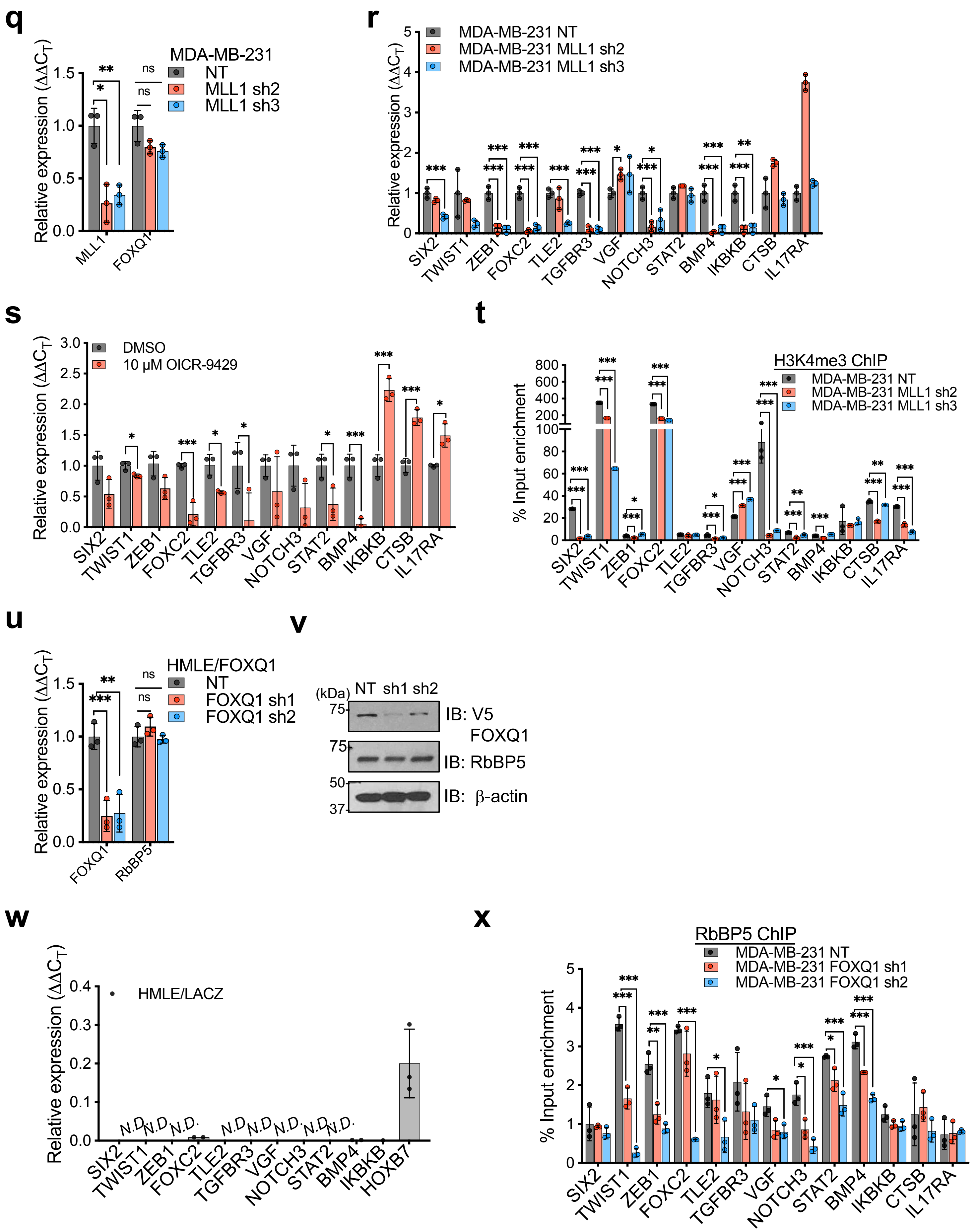

q
MDA-MB-231
NT
MLL1 sh2
MLL1 sh3
Relative expression (ΔΔC_T)
MLL1
FOXQ1
ns
r
MDA-MB-231 NT
MDA-MB-231 MLL1 sh2
MDA-MB-231 MLL1 sh3
SIX2
TWIST1
ZEB1
FOXC2
TLE2
TGFBR3
VGF
NOTCH3
STAT2
BMP4
IKBKB
CTSB
IL17RA
s
DMSO
10 μM OICR-9429
t
H3K4me3 ChIP
% Input enrichment
u
HMLE/FOXQ1
FOXQ1 sh1
FOXQ1 sh2
RbBP5
v
(kDa)
NT sh1 sh2
IB: V5 FOXQ1
IB: RbBP5
IB: β-actin
w
HMLE/LACZ
N.D.
HOXB7
x
RbBP5 ChIP
MDA-MB-231 FOXQ1 sh1
MDA-MB-231 FOXQ1 sh2

Supplementary Figure 3. RbBP5 binding within EMT promoters is dependent upon FOXQ1. (a-b) The efficiency of RbBP5 knockdown in HMLE/FOXQ1 cells was assessed via qRT-PCR (a) and western blot (b). (**c)** The knockdown efficiency of MLL1 shRNA and the effects on FOXQ1 expression in HMLE/FOXQ1 cells was assessed by qRT-PCR. (**d)** Pilot study of the effects of OICR-9429 treatment for 72 hours on H3K4me3 within the promoters of EMT transcription factors in HMLE/FOXQ1 cells. H3K4me3 ChIP-qPCR was performed to evaluated H3K4me3 occupancy within the respective EMT gene promoter regions and normalized to chromatin input samples. (**e-f)** The efficiency of FOXQ1 knockdown in MDA-MB231 cells was assessed via western blot (e) and qRT-PCR (f). (**g)** Relative expression of the FOXQ1-RbBP5 panel of EMT transcripts in MDA-MB-231 cells with shFOXQ1 or NT control. (**h-i)** The efficiency of FOXQ1 knockdown in MDA-MB468 cells was assessed via western blot (h) and qRT-PCR (i). (**j)** Relative expression of the FOXQ1-RbBP5 panel of EMT transcripts in MDA-MB-468 cells with shFOXQ1 or NT control. (**k-l)** The efficiency of RbBP5 knockdown in MDA-MB231 cells was assessed via western blot (k) and qRT-PCR (**l). (m)** Relative expression of the FOXQ1-RbBP5 panel of EMT transcripts in MDA-MB231 cells with shRbBP5 or NT control. (**n-o)** The efficiency of RbBP5 knockdown in MDA-MB468 cells was assessed via western blot (n) and qRT-PCR (o). (**p)** Relative expression of the FOXQ1-RbBP5 panel of EMT transcripts in MDA-MB-468 cells with shRbBP5 or NT control. (**q)** The efficiency of MLL1 knockdown in MDA-MB231 cells was assessed via qRT-PCR. FOXQ1 expression is not changed upon MLL1 knockdown. (**r-s)** Relative expression of the FOXQ1-RbBP5 panel of EMT transcripts in MDA-MB231 cells with shMLL1 or NT control (r) or treated with 10 µM OICR9429 for 72 hr (s). (**t)** ChIP-qPCR of H3K4me3 levels within FOXQ1 target promoters in MDA-MB-231 cells with shMLL1 or NT control. Percent input enrichment was obtained by comparing the ChIP-enriched signals to the signals obtained from the input chromatin sample. (**u-v)** The efficiency of FOXQ1 knockdown in HMLE/FOXQ1 cells assessed by qRT-PCR(u) and western block (v). (**w)** ChIP-qPCR of RbBP5 binding within FOXQ1 target promoters in HMLE/LacZ cell line. N.D. indicates DNA regions that were not detected after 60 quantitative cycles. Percent input enrichment was analyzed same as panel t.(**x)** ChIP-qPCR of RbBP5 promoter binding in MDA-MB-231 cells with FOXQ1 shRNA or NT control. Percent input enrichment was analyzed same as panel t. For q-RT-PCR experiments (panels b, c, f, g, I, j, l, m, o, p, q, r, s, u), each gene tested was normalized to β-actin in each sample and analyzed relative to the control samples. Replicate values plotted are normalized to control mean. Source data are provided in Source Data file 1. For western blots (panels a, e, k, n, v), representative images are shown (n=3). For all panels, the bar graphs indicate mean +/- SD. Individual data points mark each of the three independent replicates (n=3). P-value was calculated using unpaired, two-sided t-test with Bonferroni adjustment for multiple comparisons. * p<0.05, ** p<0.01, *** p<0.001.

Supplementary Figure 4

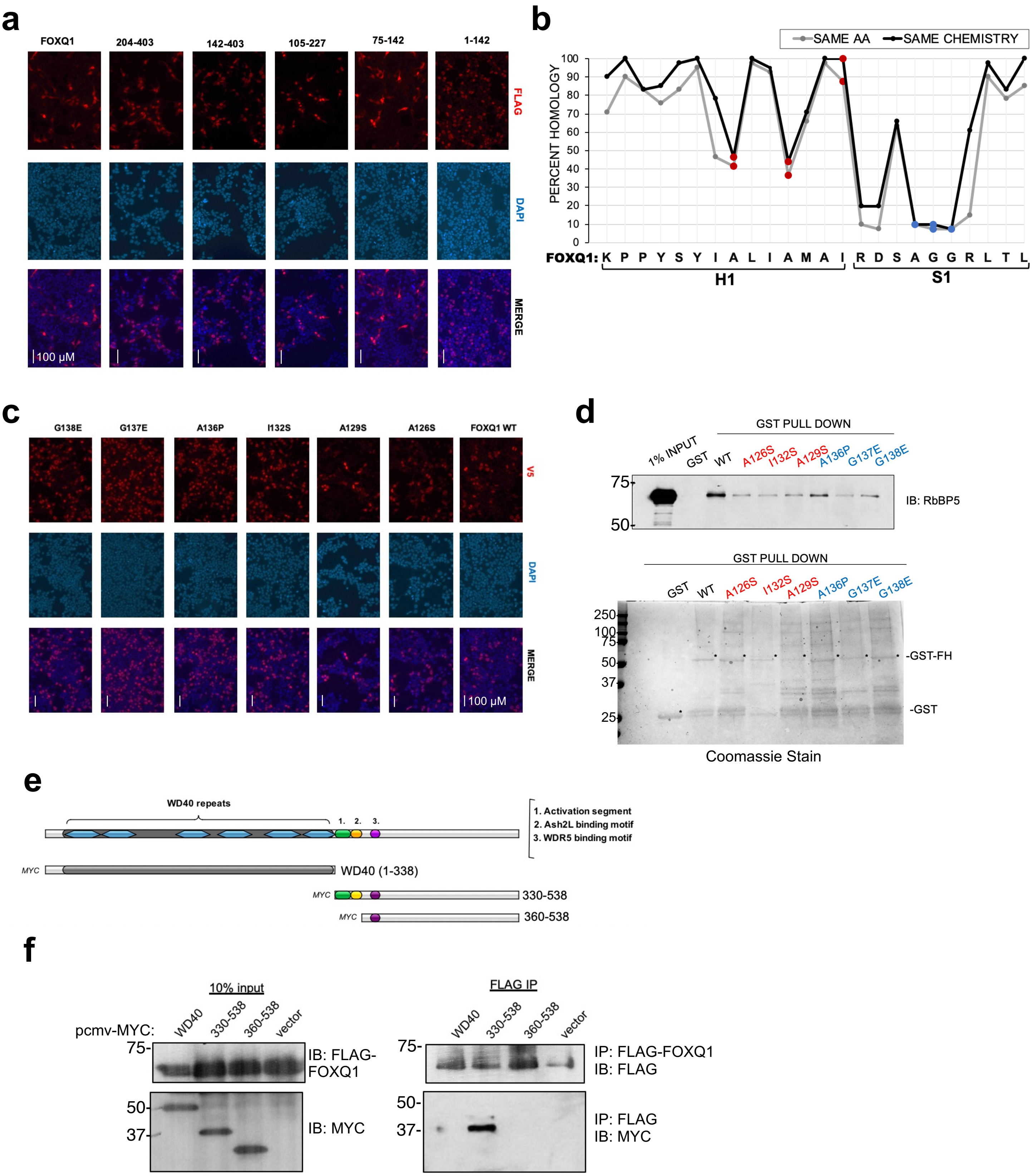

Supplementary Figure 4: Extended validation of the FOXQ1 Forkhead box binding to RbBP5. (a) Nuclear localization of all FLAG-tagged FOXQ1 fragments in HEK293 cells by immunofluorescent staining as indicated after 48 hours post transfection. Representative images (n=3). Scale bar: 100 μM. **(b)** Homology analysis of FOXQ1 human H1-S1 FHD sequence relative to the H1-S1 FHD sequences of the 50 human FOX members. Red highlights residues in H1 and blue highlights residues within S1 that are critical for RbBP5 binding. **(c)** Nuclear localization of all V5-tagged wild-type and mutated FOXQ1 with point mutations in HEK293 cells by immunofluorescent staining as indicated after 48 hours post transfection. Representative images (n=3). Scale bar: 100 μM. **(d)** GST-tagged FOXQ1-FH region (105-227), with either WT or point mutations, were purified and incubated at equimolar ratio with purified RbBP5 protein and analyzed by SDS-PAGE and western blot. GST pull-down was performed three times (n=3) and a representative result is shown. **(e)** Schematic of the RbBP5 functional domains subcloned into MYC-tag expression vector. The N-terminal contains a β-propeller domain consisting of WD40 repeats (1-338). The C-terminal of RbBP5 is unstructured and contains a central hinge region with motifs for: activating MLL (AS)(green), ASH2L binding (ABM)(yellow) and WDR5 binding (WBM)(purple). **(f)** Identification of the region of RbBP5 responsible for FOXQ1 binding by Co-IP. Full length FLAG-FOXQ1 was co-expressed with MYC-tagged RbBP5 fragments, individually, in HEK293T cells. Lysates were subject to FLAG-FOXQ1 IP and membranes were probed with MYC-antibody to detect RbBP5 fragments. Representative images are shown (n=3).

Supplementary Figure 5

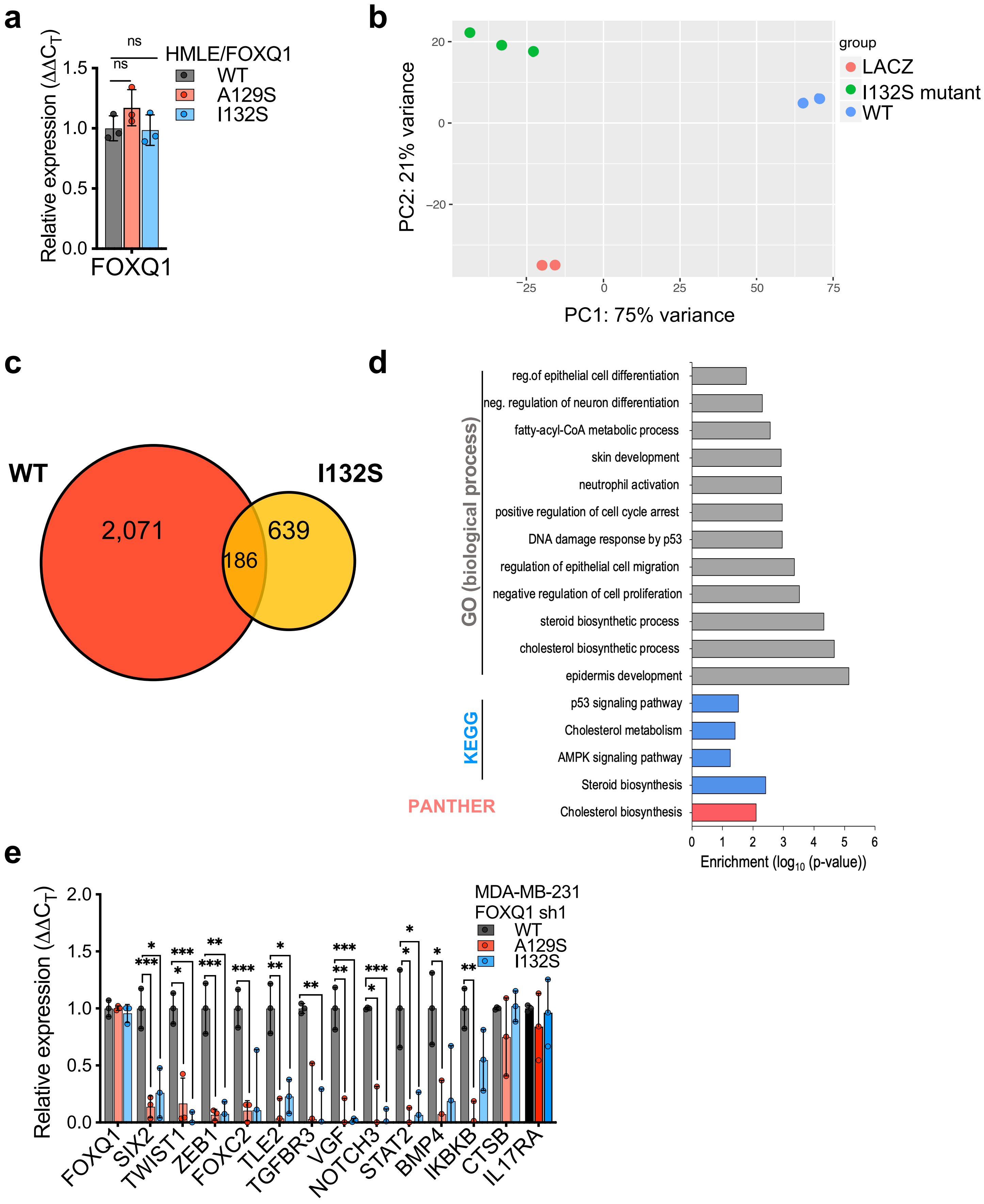

Supplementary Figure 5: Disruption of RbBP5 recruitment prevents activation of the FOXQ1 EMT program. (a) Stable lentiviral expression of FOXQ1-WT or FOXQ1 mutants (I132S, A129S) in HMLE cells was assessed by q-RT-PCR. q-RT-PCR data for FOXQ1 was normalized to β-actin in each sample and analyzed relative to the WT-FOXQ1 control cells. Bars indicate sample mean ± SD. Dots mark the values obtained from three independent replicates normalized to WT control mean (n=3). Statistical significance was assessed by two-tailed t-test with Bonferroni correction: * $p<0.05$, ** $p<0.01$, *** $p<0.001$. **(b)** Principal component analysis of the expression profiles of HMLE cells with stable FOXQ1-WT, FOXQ1-I132S or LacZ control. Each dot represents a single replicate from the RNA-seq data set (HMLE/FOXQ1-I132S n=3, HMLE/FOXQ1-WT n=2, HMLE/LacZ n=2). **(c)** Overlap of the differentially downregulated gene set in HMLE/FOXQ1-WT or HMLE/FOXQ-I132S mutant cell line relative to HMLE/LacZ identified by RNA-seq. (d) Functional enrichment analysis of the genes upregulated in HMLE/FOXQ1 I132S mutant cells relative to HMLE/LacZ using Enrichr. Adjusted p-value was derived by comparing the gene set against the indicated annotation tools by Fisher's exact t-test with Benjamini-Hochberg method of correction for multiple hypotheses testing. Bar graph plots the $\log_{10}$ adjusted p-value (Enrichment). **(e)** Expression of FOXQ1-regulated EMT target genes in MDA-MB-231 FOXQ1-sh1 cells with rescued expression of FOXQ1 mutants, A129S or I132S, or WT control. q-RT-PCR data for each gene was normalized to β-actin in each sample and analyzed relative to the WT-FOXQ1 cells. Bars indicate sample mean ± SD. Dots mark the values obtained from three independent replicates normalized to the control mean (n=3). Statistical significance was assessed by unpaired, two-tailed t-test: * $p<0.05$, ** $p<0.01$, *** $p<0.001$. For panels a and e, Source data are provided in Source Data 1.

Supplementary Figure 6

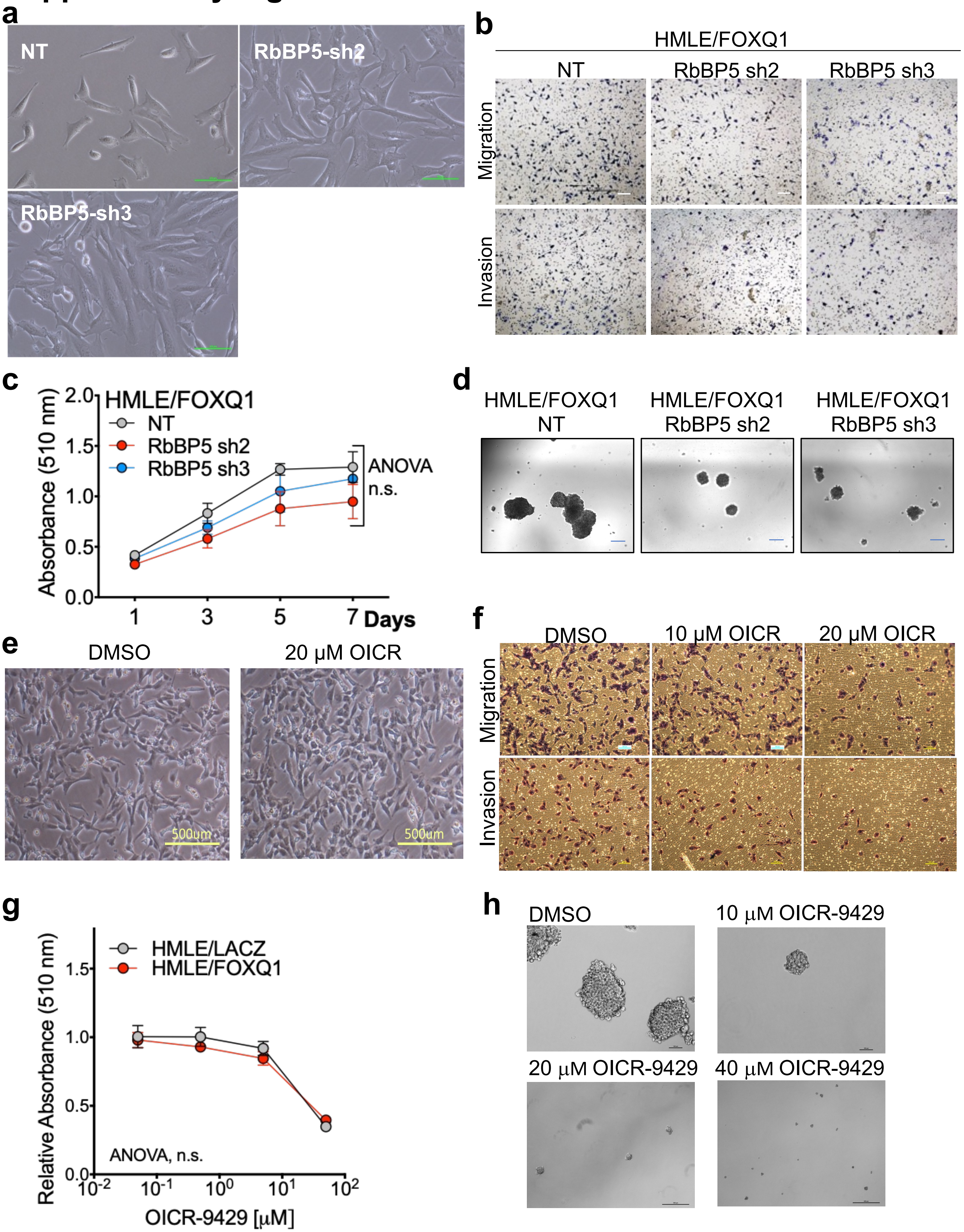

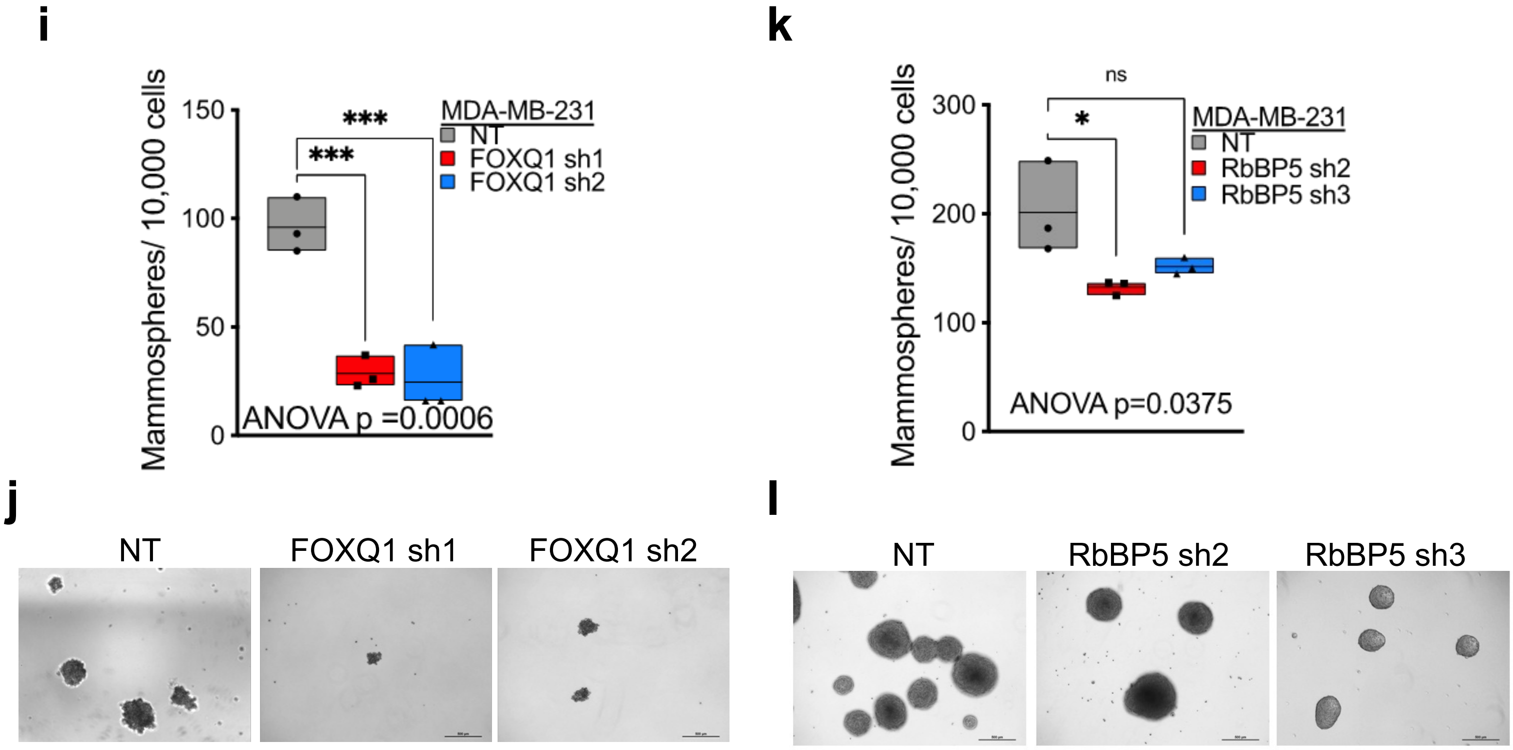
i
Mammospheres/ 10,000 cells
150
100
50
0
***
***
MDA-MB-231
NT
FOXQ1 sh1
FOXQ1 sh2
ANOVA p =0.0006
k
Mammospheres/ 10,000 cells
300
200
100
0
ns
*
MDA-MB-231
NT
RbBP5 sh2
RbBP5 sh3
ANOVA p=0.0375
j
NT
FOXQ1 sh1
FOXQ1 sh2
l
NT
RbBP5 sh2
RbBP5 sh3

Supplementary Figure 6: Targeting the MLL core complex decreases the FOXQ1-driven oncogenic phenotype *in vitro.* (a) Representative pictures show morphologic change after knockdown of RbBP5 in HMLE/FOXQ1 cells. Scale bar: 100 µm. Representative images (n=3). (**b)** Visualization of migration (top panels) and invasion (bottom panels) inserts of HMLE/FOXQ1 cells with shRbBP5 or NT control. Representative images are shown (n=3). Scale bar: 100 µm. **(c)** Sulforhodamine B colorimetric (SBC) proliferation assay of HMLE/FOXQ1 shRbBP5 knockdown or NT control cells. Absorbance at 510nm, as a proxy for cell abundance, was measured at indicated time points. Mean absorbance ± SD results are shown (n=6). Data were analyzed by repeated measures one-way ANOVA with Bonferroni correction. **(d)** Images of mammospheres formed by HMLE/FOXQ1 NT and shRbBP5 knockdown cells. Representative images are shown (n=3). Scale bar: 500 µm. **(e)** Representative pictures show morphologic change after treatment of 20 µM OICR -9429 in HMLE/FOXQ1 cells 72 hours post treatment. Representative images are shown (n=3). Scale bar: 500 µm. (**f)** Visualization of migration (top panels) and invasion (bottom panels) inserts from HMLE/FOXQ1 cells treated with 10 and 20 µM OICR-9429 or DMSO control. Representative images are shown (n=3). Scale bar: 100 µm. (**g)** Effects of OICR-9429 treatment on cell proliferation analyzed by SBC assay. Absorbance at 510nm was measured at indicated time points and normalized to the DMSO mock treatment group for each cell line. Mean absorbance ± SD results are shown (n=6). Data were analyzed by two-way ANOVA with Bonferroni correction for multiple comparisons. (**h)** Images of mammospheres formed by HMLE/FOXQ1 cells pretreated with indicated doses of OICR-9429 for 72 hours prior to plating. Representative images are shown (n=3). Scale bar: 500 µm. (**i-j)** Quantification **(i)** and visualization **(j)** of mammospheres formed by MDA-MB-231 cells with stable FOXQ1 shRNA knockdown or NT control. Scale bar: 500 µm. (**k-l)** Quantification **(k)** and visualization **(l)** of mammospheres formed by MDA-MB-231 with stable RbBP5 shRNA knockdown or NT control. Scale bar: 500 µm. For panels i-l: Mammospheres ≥ 50 µm in diameter were quantified. Floating bars mark the sample mean, minimum and maximum values. Data were normalized to the mean of the control group. Dots mark the individual values from three individual replicates (n=3). Groups were compared by one way ANOVA with Bonferroni correction for multiple comparisons. * p<0.05, ** p<0.01, *** p<0.001.

Supplementary Figure 7

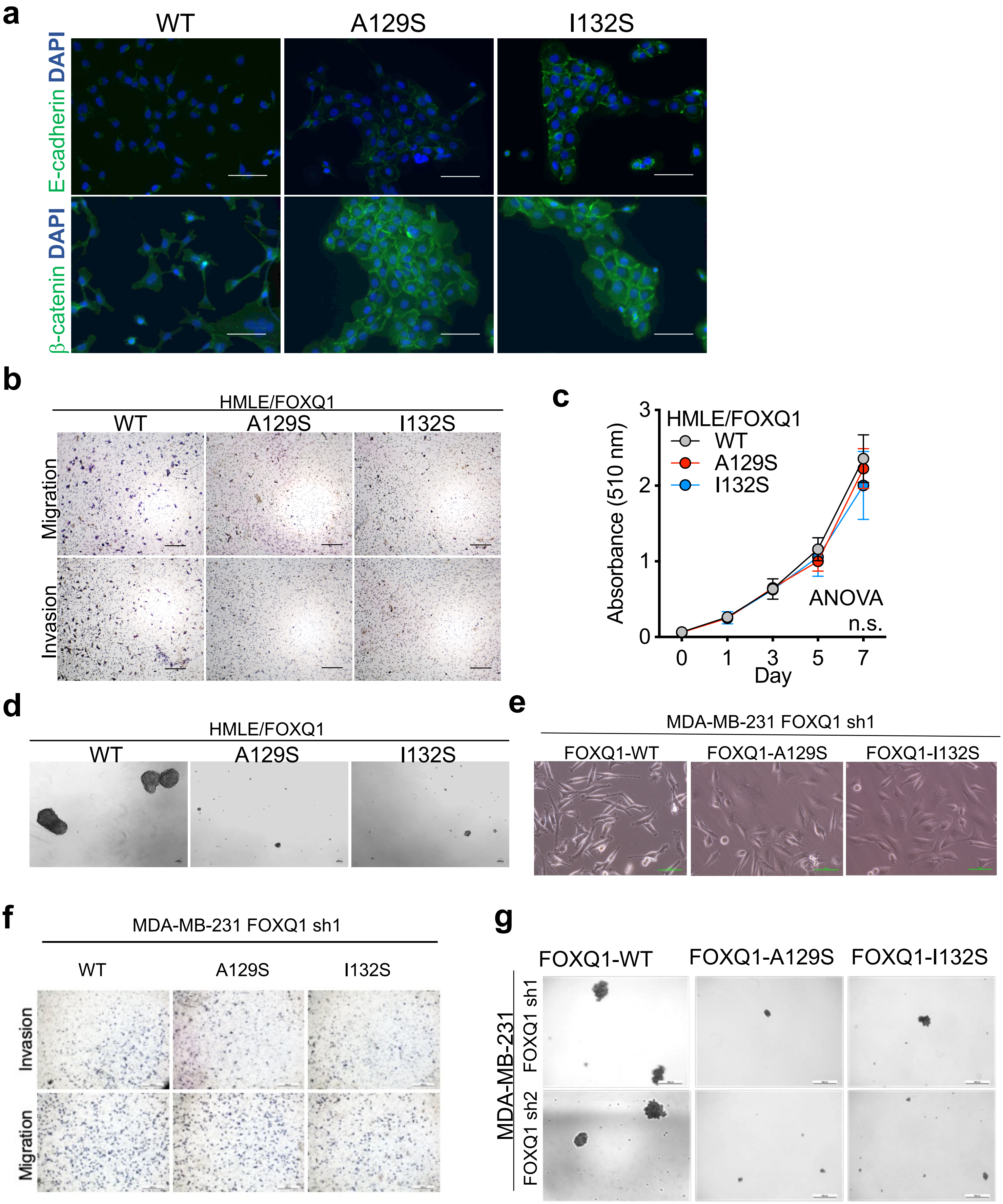

Supplementary Figure 7: Expression of FOXQ1 RbBP5-defective mutants results in a decrease in FOXQ1-driven oncogenic potential *in vitro.* **(a)** Immunofluorescence staining of E-cadherin (top panels) and β-catenin (bottom panels) localization in HMLE/FOXQ1-WT, HMLE/FOXQ1-A129S, and HMLE/FOXQ1-I132S cells**.** Representative images (n=3). Scale bar: 100 µM. **(b)** Visualization of cells migrated (top panels) and invaded (bottom panels) in WT and mutant FOXQ1 HMLE cell models. Representative field images are shown (n=3). Scale bar: 100 µm. **(c)** Cell proliferation of HMLE cells with WT, A129S or I132S FOXQ1 expression measured by SBC assay. Absorbance at 510 nm measured at indicated time points. Mean absorbance ± SD shown from 6 independent replicates (n=6). Data were analyzed by repeated measures one-way ANOVA with Bonferroni correction. **(d)** Visualization of the mammosphere formation capability of HMLE/FOXQ1-WT, HMLE/FOXQ1-A129S, and HMLE/FOXQ-I132S cells. Representative field images are shown (n=3). Scale bar: 100 µm. **(e)** Morphology of MDA-MB-231shFOXQ1 cells expressing either WT, A129S, or I132S FOXQ1**.** Representative images are shown (n-3)**.** Scale bar: 100 µm. **(f)** Visualization of migration (top panels) and invasion (bottom panels) inserts from MDA-MB-231shFOXQ1 cells expressing either WT, A129S, or I132S FOXQ1. Representative field images are shown (n=3). Scale bar: 100 µm. **(g)** Visualization of the mammosphere formation capability of MDA-MB-231shFOXQ1 cells expressing either WT or mutant (A129S, I132S) FOXQ1. Representative field images are shown (n=3). Scale bar: 500 µm. For panels c, g, i and k, source data are provided in Source Data file 1.

Supplementary Figure 8

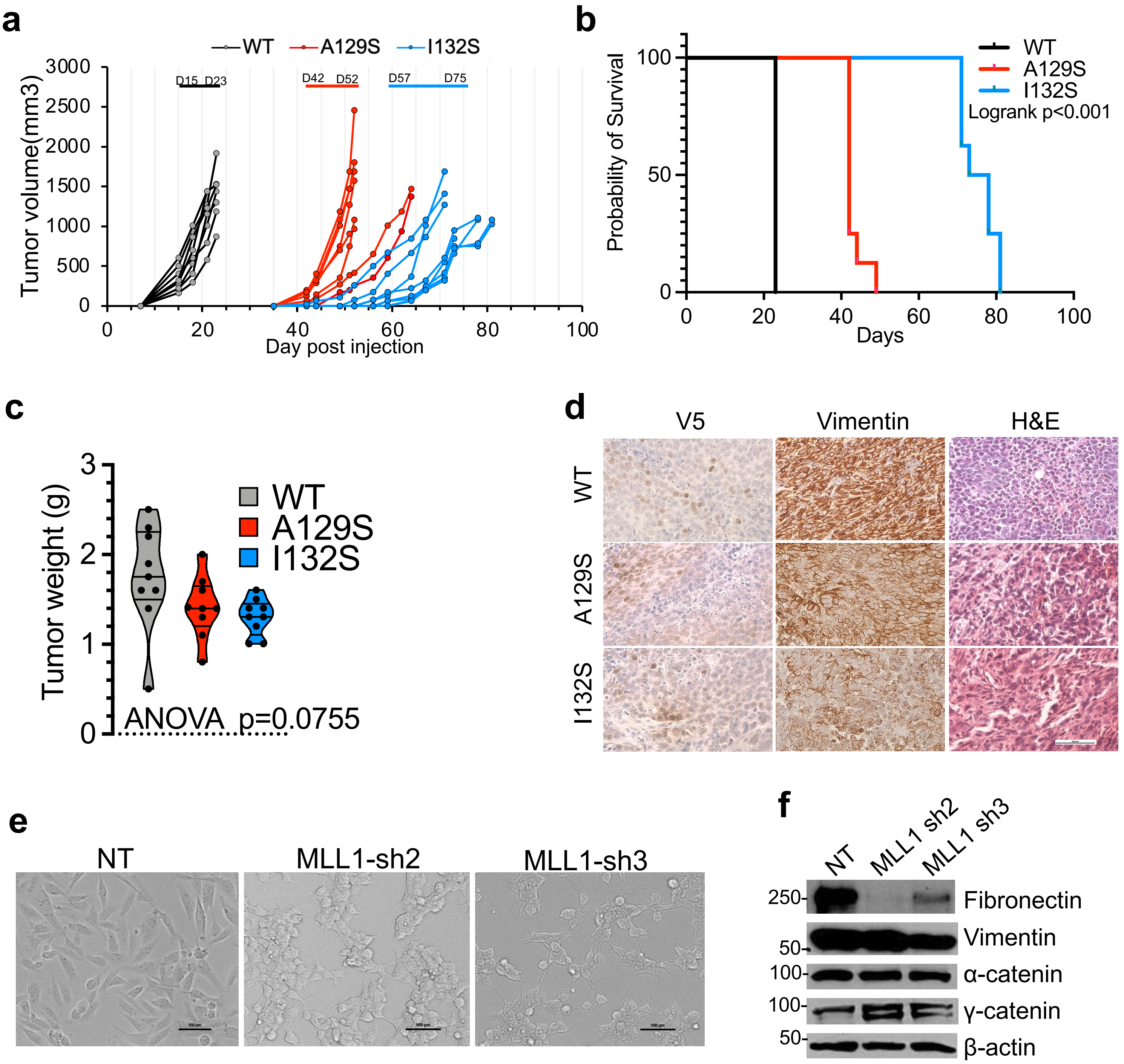

Supplementary Figure 8: Disruption of the FOXQ1-RbBP5 interaction results in an inhibition of tumor progression *in vivo.* (**a)** Results of tumor formation and growth of HMLER/FOXQ1-WT, HMLER/FOXQ1-A129S, and HMLER/FOXQ1-I132S cells engrafted orthotopically into mammary fat pads of NSG mice. Mice were sacrificed when tumor reached approximately 1500 mm^3 with n=8 mice per group. Tumor volume was measured approximately every three days by caliber. The median tumor onset and median endpoint are also marked for each group as timeline above the line graph. (**b)** Kaplan Meyer plot of the time to euthanasia of NSG mice engrafted with HMLER cells expressing either FOXQ1-WT, FOXQ1-A129S, or FOXQ1-I132S (n=8 mice per group). Survival analysis of the time to euthanasia for NSG mice engrafted with HMLER cells expressing either FOXQ1-WT, FOXQ1-A129S, or FOXQ1-I132S (n=8 mice per group) by log rank test. (**c)** Summary of the final tumor weights for the primary tumors formed from the engraftment of HMLER cells with indicated FOXQ1 expression (n=8 mice per group). Data were analyzed by one-way ANOVA with Bonferroni correction for multiple comparisons. (**d)** IHC staining of the primary tumors formed from the engraftment of HMLER cells. expressing either WT-, A129S-, or I132S- FOXQ1, for vimentin expression and V5-antibody detection of tagged FOXQ1 protein. The image is representative of IHC staining from each of the primary tumors (n=5 per tumor). Scale bar:100 μm. **(e)** Representative images of cell morphology for MDA-MB231 cells with and without MLL1 knockdown. Representative images (n=3). Scale bar:100 μm. **(f)** Western blotting analysis of EMT markers in MDA-MB231 cells with MLL1 knockdown and non-target cells. Representative images (n=3). For panels a, b and c, source data are provided in Source Data file 1.

Supplementary Table 1: Sequences of gene-specific shRNAs

shRNA	Clone ID	Targeting sequence (5'→3')
RbBP5 sh2	TRCN0000034413	GCCTTCTGTAGCAGTGATGAA
RbBP5 sh3	TRCN0000034410	GCTCTATTGTATTTACCCATT
FOXQ1 sh1	V3LHS_381794	GAGCATCCAGTAGTTGTCC
FOXQ1 sh2	V3LHS_381789	TGGGGTTGAGCATCCAGTA
MLL1 sh2	TRCN0000005955	ATACTTCTGGTTCTGGATGGG
MLL1 sh3	TRCN0000005956	AAATGAGAGCTGCTTTAGGCG

Note: pLKO.1 vector expressing scramble shRNA (designated as NT) was used to match RbBP5 and MLL1 shRNA. pGipZ vector expressing scramble shRNA (designated as NT) was used to match FOXQ1 shRNA.

Supplementary Table 2: PCR primers, Cloning primers and mutagenesis primers

Quantitative reverse-transcriptase PCR primers

Gene	Forward Primer (5'→3')	Reverse Primer (5'→3')
FOXQ1	AACGACTGCTTCGTCAAGGT	GCATCCAGTAGTTGTCCTTGC
RbBP5	CCAAGAAGAAGCAAGCAGGC	ATTGCTCCTCCTGCTGTCAA
SIX2	GCCTGCGAGCACCTCCACAAGAAT	CACCGACTTGCCACTGCCATTGAG
TWIST1	TCGGTCTGGAGGATGGAGGGG	AATGACATCTAGGTCTCCGGCCC
ZEB1	TGACCTGCCAACAGACCAGACA	CCTTTCCTGTGTCATCCTCCCAGC
FOXC2	GCTCGCTCAGGTAGGGCACC	CGAGCCGTCTCGGAAGCAGC
TLE2	CTGTCCCCTGAAGTTTGCCTC	GGACTGAGGACGACTCCTTG
TGFBR3	GATGGCGTAGTTTTGCCGC	GCAATTTTCAAACTGCCTCGGA
VGF	TCAGTCCAAGTAGCGCCAAG	GGCTCTCCAGATTCACTCGG
NOTCH3	GAGCCAATGCCAACTGAAGAG	GGCAGATCAGGTCGGAGATG
STAT2	GGTGTGGAAGCTTGGTGTGA	AGGAGGAGAACTGCCAACTG
BMP4	CACCTCATCACACGACTACT	TCCAGTATCCCCAAAGCCTGT
BMP4?	CGTCCAAGCTATCTCGAGCC	CGGAATGGCTCCATAGGTCC
IKBKB	GATTGCCATCAAGCAGTGCC	GGGTGGGTCAGCCTTCTCAT
CTSB	CTCCTGCTGGCTGTAATGGT	GGATGGAGTACGGTCTGCAC
IL17RA	AGAACCAATTCCGGGGCCT	GGTCGGCTGAGTAGATGATCC
HOXB7	CCCTTTGAGCAGAACCTCTCC	CGGTCAGTTCCTGAGCTTCG
β-ACTIN	CAGAAGGAGATCACTGCCC	CATCTGCTGGAAGGTGGAC

Quantitative ChIP PCR primers

Promoter	Forward Primer (5'→3')	Reverse Primer (5'→3')	Amplicon chromosomal location (hg19)
SIX2	ACTCGGAGAGGAGAAGAAACT	GCTTTCTCGGGCTATGCACT	chr2: 45255184-45255278
TWIST1	CTGCAGACTTGGAGGCTCTT	CTCACGTCAGGCCAATGACA	chr10: 31598478 -31598586
ZEB1	ACTGTCCCATTTGCCCTTCATA	AGCATTGTCTGATGGGAAGTACA	chr7: 19157269-19157344
FOXC2	CTACAGCTACATCGCGCTCA	TCTCCCCGGTAGAAGGGGAAG	chr16: 86601169-86601275
TLE2	GCTATTATCACTGGGCTACCTGG	GGCAAGCTCTCAGCCCCATAA	chr19: 3041332-3041407
TGFBR3	TGGATGCTTCAAACAGGCTCT	GGACAGGAGAAACTCATTCCT	chr1: 92115428-92115518
VGF	TCTCCCCCACCCCAAAATTC	ATCCAATGGGATCAGTTTGCC	chr7:100825337-100825441
NOTCH3	TGACACAGGCGTCATCCAG	CAAGGGGTGTGGGCCTTTT	chr19:15300207-15300306
STAT2	GGAGTCCCTTTGGAAGTTTGGT	CACTAGCCACTTCCCCTCATC	chr12: 56734932-56735013
BMP4	ACCAGGAACTTTGGTGTCCA	TGTGGGTCTCAGTCTCATCTG	chr14: 54433720-54433803
IKBKB	AGCTCCAGGTTGTACAGAAAACA	TCACCTGATGGCAGTGAAGC	chr8: 42123850-42123928
CTSB	TGCTCATTAGTGTTCTGCCTCT	TAATCTCGCCTGCGGTTGAA	chr8: 11719310-11719380
IL17RA	GGGCAGAGGCCTTTCTCATTC	TCTGAGCTCCTAGCTCTTAGGG	chr22: 17564162-17564230
HOXB7	GTTCCCAGGGGTACTGAGTG	CTTTGATTTGGAGGGGCCGA	chr17: 46678594-46678686

FOXQ1 site directed mutagenesis primers (pENTR)

Mutation	Forward Primer (5'→3')	Reverse Primer (5'→3')
K112E	AGGGTGCACGCAGCGAGCCATATACGC	GCGTATATGGCTCGCTGCGTGCACCCT
A126S	CCCTACTCGTACATCAGCCTCATCGCCATGGCCATCCG	CGGATGGCCATGGCGATGAGGCTGATGTACGAGTAGGG
I128S	TCATCGCCATGGCCAGCCGCGACTCGGC	GCCGAGTCGCGGCTGGCCATGGCGATGA
A129S	TCGTACATCGCGCTCATCTCCATGGCCATCCGC	GCGGATGGCCATGGAGATGAGCGCGATGTACGA
A136P	ATCCGCGACTCGCCGGGCGGGCGCTTGACG	CGTCAAGCGCCCGCCCGGCGAGTCGCGGAT
G137E	ATCCGCGACTCGGCGGAAGGGCGCTTGACGCTGGCG	CGCCAGCGTCAAGCGCCCTTCCGCCGAGTCGCGGAT
G138E	CGACTCGGCGGGCAAGCGCTTGACGCTGGCGGA	TCCGCCAGCGTCAAGCGCTTGCCCGCCGAGTCG
R164E	CAGCTACACGGGCTGGGAAAACTCCGTGCGCCACAACC	GGTTGTGGCGCACGGAGTTTTCCCAGCCCGTGTAGCTG
R168E	GGCTGGCGCAACTCCGTGGAACACAACCTTTCGCTCAA CG	CGTTGAGCGAAAGGTTGTGTTCCACGGAGTTGCGCCAGC C

Cloning Primers

FOXQ1 fragment cloning (pcmv7.1-3xFLAG)

Region (AA position)	Forward Primer (5'→3')	Reverse Primer (5'→3')
1-142	AGGGTGCACGCAGCGAGCCATATACGC	GCGTATATGGCTCGCTGCGTGCACCCT
105-227	CCCTACTCGTACATCAGCCTCATCGCCATGGCCATCCG	CGGATGGCCATGGCGATGAGGCTGATGTACGAGTAGGG
205-403	TCATCGCCATGGCCAGCCGCGACTCGGC	GCCGAGTCGCGGCTGGCCATGGCGATGA
143-403	TCGTACATCGCGCTCATCTCCATGGCCATCCGC	GCCGAGTCGCGGCTGGCCATGGCGATGA
75-142	ATCCGCGACTCGCCGGGCGGGCGCTTGACG	GCGTATATGGCTCGCTGCGTGCACCCT

FOXQ1 GST cloning (pGEx-6p2)

FOXQ1	Forward Primer (5'→3')	Reverse Primer (5'→3')
Full Length	GAAGCGGATCCATGAAGTTGGAGGTGTTCGT	CCTCGACTCGAGTCAGGCTAGGAGCGTCTCC
105-227	CCCTACTCGTACATCAGCCTCATCGCCATGGCCATCCG	CGGATGGCCATGGCGATGAGGCTGATGTACGAGTAGGG

RbBP5 fragment cloning (pcmv-MYC)

Region (AA position)	Forward Primer (5'→3')	Reverse Primer (5'→3')
1-338	TGGCCATGGAGGCCCGAATTCGGATGAACCTCGAGTTG CTGG	GGATCCCCGCGGCCGCGGTACCTCATTCTTTGAAGTCTG GTGCAAAT
330-538	TGGCCATGGAGGCCCGAATTCGGAGTGCATTTGCACCA GACT	GGATCCCCGCGGCCGCGGTACCTCATAACAGTTCTGA GATTGCT
360-538	TGGCCATGGAGGCCCGAATTCGGAGTGAGCCTGAGCAG ACAG	GGATCCCCGCGGCCGCGGTACCTCATAACAGTTCTGA GATTGCT

Supplementary Table 3: Antibodies

Antibody	Species	Clone	Lot. No	Supplier	Catalog No.	WB	IF	FACs	IP
anti-RbBP5	Rabbit		3	Bethyl	A300-109A	1:1000			1:200
anti-V5	Mouse		2212258	invitrogen	46-0705	1:1000	1:800		1:200
Anti-H3K4me3: ChIPAb+ Trimethyl-Histone H3 (Lys4)	Rabbit		2871690	EMD Millipore	17-614				1:100
Anti-FOXQ1 antigen: KLEVFVPRAAHGD KQGSDLEGAGGSD APSPL	Rabbit			Dr. Shengping Zhang and Dr. Chuanggui Wang	N/A	1:3000			
anti-ASH2L	mouse		H3117	Santa Cruz Biotechnology	sc-81184	1:1000			
anti-WDR5	Rabbit		1	Bethyl	A302-430A	1:1000			
anti-β-actin (C4)	Mouse	C4	E0720	Santa Cruz Biotechnology	sc-47778	1:2000			
anti-N-Cadherin	mouse	32/N-cadherin	9322775	BD Transduction Labs	610920	1:2000			
anti-Vimentin	Rabbit		8	Cell Signaling Technology	5741S	1:1000			
anti-Fibronectin	Mouse	10/Fibronect in	9070804	BD Transduction Labs	610077	1:5000			
anti-Claudin-1 (D-4)	mouse	D-4	D1218VL	Santa Cruz Biotechnology	sc-137121	1:500			
Anti-Occludin	mouse	E5	JO118	Santa Cruz Biotechnology	sc-133256	1:500			
anti-FLAG	mouse		SLBT7654	Sigma Aldrich	F1804 0.2 MG		1:800		
Anti-Myc	Rabbit		5	Cell Signaling Technology	2278S	1;1000	1:400		
anti-HA	Mouse	2-2.2.14	RJ241582	invitrogen	26183	1:5,00 0			
anti-E-cadherin	mouse	36/E-cadherin	1033217	BD Transduction Labs	610405	1:2000			
anti-α-catenin	mouse	5/a-catenin	31292	BD Transduction Labs	610193	1:500			
anti-β-catenin	mouse	14/Beta-catenin	20079	BD Transduction Labs	610153	1:1000			
anti-γ-catenin	mouse	15/γ-Catenin	15770	BD Transduction Labs	610253	1:2000			
Anti-KMT2A/MLL1	Rabbit		5	Bethyl Laboratory	A300-374A	1:2000			
Anti-KMT2B/MLL2	Rabbit		VL314831 8	Invitrogen	PA5-103371	1:1000			
Anti-KMT2C/MLL3	Rabbit		129K0565	SIGMA-ALDRICH	SAB130008 2	1:1000			
Anti-KMT2D/MLL4	Rabbit		3487515	EMD millipore	ABE1867	1:500			
Anti-KMT2E/SET1A	Rabbit		7	Bethyl laboratory	A300-289A-M	1:1000			
Anti-KMT2F/SET1B	Rabbit		1	Bethyl laboratory	A302-280A	1:500			
Horse Anti-Mouse IgG Antibody (H+L), Peroxidase	Mouse		ZG1208	Vector Laboratories	PI-2000-1	1:3000			
anti-Rabbit IgG horse radish peroxidase linked	Rabbit		27	Cell Signaling Technology	7074	1:3000			
PE anti-Human CD24	Mouse	ML5	5049759	BD Pharmingen	555428			1:100	
FITC anti-Human CD44	Mouse	G44-26	5275777	Pharmingen	555478			1:100	
Alexa Fluor 488 goat anti-mouse IgG	Mouse		481679	invitrogen	A11001		1:800		
Alexa Fluor 594 goat anti-mouse IgG	Mouse		610868	invitrogen	A11005		1:800		
