# Supplementary material for: FOXQ1 recruits the MLL complex to activate transcription of EMT and promote breast cancer metastasis
Source: Nat Commun. 2022 Nov 1;13:6548. doi: 10.1038/s41467-022-34239-z (PMC9626503; doi:10.1038/s41467-022-34239-z)
Supplement: Supplementary file 3 — Description of Additional Supplementary Files [file 41467_2022_34239_MOESM3_ESM.pdf]

Description of Additional Supplementary Files

File Name: Supplementary Data 1
Description: **FOXQ1 (vs. LACZ vector control) Tandem Affinity Purification Mass Spectrometry to identify candidate interacting proteins**
TAP-TAG Purification System
Full-length wild-type human FOXQ1 gene was cloned into pcDNA3.1(C)-STAP vector. The resultant TAP-tag consists of streptavidin binding protein (SBP) and protein A, separated by a TEV protease site, fused to the C-terminus of FOXQ1. A construct expressing LacZ TAP-tag was used as the control.
Mass Spectrometry Protein Identification
The Uniprot_Hum_Compl_20150826 database was searched for human protein sequences, and a reverse decoy protein database was run simultaneously for false discovery rate (FDR) determination. Secondary analysis was performed using Scaffold 4.4.5 (Proteome Software). Minimum protein identification probability was set at two unique peptides with ≤ 1.0% FDR.
Data set
A total of 510 proteins were identified from over 2500 spectra. The resulting proteins identified in the FOXQ1 TAP samples and LacZ negative control were compared based on the number of peptides mapped to the protein ID, normalized to the overall protein molecular weight by Fischer's exact T-test

File Name: Supplementary Data 2
Description: **HMLE/FOXQ1-WT (vs. LACZ vector control) RNA-seq differential expression analysis**
Analysis
Sequencing was performed using the Illumina HiSeq 2000 platform with 100 bp paired-end reads on-average min and of about 40-50 million reads per sample. Paired-end reads were mapped to the hg19 human genome using Bowtie2 v2.2.9. The abundance was estimated using RSEM. Genes with < 10 counts were filtered. Differental expression analysis was done using EdgeR v3.12.1 in the Bioconductor package.

File Name: Supplementary Data 3
Description: **HMLE/FOXQ1- I132S mutant (vs. LACZ vector control) RNA-seq differential expression analysis**
Analysis
Sequencing was performed using the Illumina HiSeq 2000 platform with 100 bp paired-end reads on-average min and of about 40-50 million reads per sample. Paired-end reads were mapped to the hg19 human genome using Bowtie2 v2.2.9. The abundance was estimated using RSEM. Genes with < 10 counts were filtered. Differental expression analysis was done using EdgeR v3.12.1 in the Bioconductor package.
