# Supplementary material for: FOXQ1 recruits the MLL complex to activate transcription of EMT and promote breast cancer metastasis
Source: Nat Commun. 2022 Nov 1;13:6548. doi: 10.1038/s41467-022-34239-z (PMC9626503; doi:10.1038/s41467-022-34239-z)
Supplement: Supplementary file 7 — Reporting Summary [file 41467_2022_34239_MOESM7_ESM.pdf]

nature portfolio

Corresponding author(s): Guojun Wu

Last updated by author(s): Oct 5, 2022

Reporting Summary

Nature Portfolio wishes to improve the reproducibility of the work that we publish. This form provides structure for consistency and transparency in reporting. For further information on Nature Portfolio policies, see our Editorial Policies and the Editorial Policy Checklist.

Statistics

For all statistical analyses, confirm that the following items are present in the figure legend, table legend, main text, or Methods section.

n/a	Confirmed	
☐	☒	The exact sample size (*n*) for each experimental group/condition, given as a discrete number and unit of measurement
☐	☒	A statement on whether measurements were taken from distinct samples or whether the same sample was measured repeatedly
☐	☒	The statistical test(s) used AND whether they are one- or two-sided<br>*Only common tests should be described solely by name; describe more complex techniques in the Methods section.*
☒	☐	A description of all covariates tested
☐	☒	A description of any assumptions or corrections, such as tests of normality and adjustment for multiple comparisons
☐	☒	A full description of the statistical parameters including central tendency (e.g. means) or other basic estimates (e.g. regression coefficient) AND variation (e.g. standard deviation) or associated estimates of uncertainty (e.g. confidence intervals)
☐	☒	For null hypothesis testing, the test statistic (e.g. *F*, *t*, *r*) with confidence intervals, effect sizes, degrees of freedom and *P* value noted<br>*Give P values as exact values whenever suitable.*
☒	☐	For Bayesian analysis, information on the choice of priors and Markov chain Monte Carlo settings
☒	☐	For hierarchical and complex designs, identification of the appropriate level for tests and full reporting of outcomes
☒	☐	Estimates of effect sizes (e.g. Cohen's *d*, Pearson's *r*), indicating how they were calculated

Our web collection on statistics for biologists contains articles on many of the points above.

Software and code

Policy information about availability of computer code

Data collection	RNA sequencing data was collected by Illumina HiSeq 2000 platform. ChIP-seq data was collected Illumina 400 platform. Flow data was collected by BD FACS Diva 4.0 software. Proteomics Data was collected by Orbitrap Fusion™ Tribrid mass spectrometer with Xcalibur to operate the instrument (Thermo)

Data analysis	ChIP-qPCR/q-RT-PCR: The data were analyzed in Microsoft Excel (Version 16.40) and Prism 8 (Version 8.4.3). P-values were calculated by unpaired two-sided t-test. For >2 samples, multiple comparison was made to the respective control group and p-value was adjusted by Bonferroni correction.<br>ChIP-seq: The data were analyzed on Galaxy (https://usegalaxy.org/), an open-soure web-based platform. Reads were mapped using Bowtie2 (Version 2.3.2.2) using the built-in Homo sapiens (b37): hg19 reference genome. ChIP-seq peaks were called from alignment results for each biological replicate using MACS2 (Galaxy Version 2.1.1.20160309.0) relative to input, control sample. Peak detection was based on FDR (qvalue) set to 0.001. The resulting bedgraph files were converted to bigwis using 'Wig/BedGraph-to-bigWig converter' (Galaxy Version 1.1.1). Enrichment on chromosome and annotation (CEAS) was conducted on peak BED files using Galaxy/Cistrome (https://cistrome.org/ap) CEAS version 1.0.0. Motif analysis was conducted using peak summits submitted to MEME Suite (Version 5.4.4) at http://meme-suite.org/tools/meme-chip.<br>RNA-seq: Data was analyzed using R Studio (Version 1.2.5033) and the Bioconductor package (Version 3.1.0). Paired-end reads were mapped to the hg19 human genome using Bowtie2 v2.2.9. The abundance was estimated using RSEM and the differential expression analysis was done using EdgeR v3.12.1 in the Bioconductor package.<br>Proteomics Data analysis was performed first with Proteome Discoverer 1.4 (Thermo). Secondary analysis was performed using Scaffold 4.4.5 (Proteome Software).<br>Flow data were analyzed on FlowJo v10 software.

For manuscripts utilizing custom algorithms or software that are central to the research but not yet described in published literature, software must be made available to editors and reviewers. We strongly encourage code deposition in a community repository (e.g. GitHub). See the Nature Portfolio guidelines for submitting code & software for further information.

Data

Policy information about availability of data

All manuscripts must include a data availability statement. This statement should provide the following information, where applicable:

- Accession codes, unique identifiers, or web links for publicly available datasets
- A description of any restrictions on data availability
- For clinical datasets or third party data, please ensure that the statement adheres to our policy

The source data underlying Figs. 2f, 3a–h, 5b, 5d-f, 6a-b, 6d, 6f-g, 6i, 7b-d, 7g-j, 8a, 8c ,8e, 8g and Supplementary Figs. 3a, 3c-d, 3f-g, 3i-j, 3l-m, 3o-p, 3q-r, 3s, 3t, 3u, 3x, 5a, 5d-e, 6c, 6g, 6i, 6k, 7c, 8b-c are provided as a Source data 1. Unprocessed original scans of blots are shown in Source data 2. The remaining data are contained within the Supplementary Information or are available from the authors upon request. The Uniprot_Hum_Compl_20150826 database https://www.uniprot.org/uniparc?query=(dbid:20150826) was searched for human protein sequences in this study. The RNA-sequencing and ChIP-sequencing data in this study have been deposited into the National Center for Biotechnology Information (NCBI) Gene Expression Omnibus (GEO) database with the accession code GSE141293. A reporting summary for this article is available as a Supplementary Information file.

Human research participants

Policy information about studies involving human research participants and Sex and Gender in Research.

Reporting on sex and gender	N/A
Population characteristics	N/A
Recruitment	N/A
Ethics oversight	N/A

Note that full information on the approval of the study protocol must also be provided in the manuscript.

Field-specific reporting

Please select the one below that is the best fit for your research. If you are not sure, read the appropriate sections before making your selection.

☒ Life sciences ☐ Behavioural & social sciences ☐ Ecological, evolutionary & environmental sciences

For a reference copy of the document with all sections, see nature.com/documents/nr-reporting-summary-flat.pdf

Life sciences study design

All studies must disclose on these points even when the disclosure is negative.

Sample size	For in vitro studies, sample sizes (n=/>3). Sample size as estimated according to previous successful experience and to be large enough to obtain reproducible results.<br>For in vivo studies, our prior studies have found an average of ~60 lung lesions per mouse in MDA-MB-231 xenograft mouse model with a standard deviation of 5. A sample size of 8 animals per group was selected and was determined to be sufficient to detect a difference of 1

	standard deviation units at 0.95 based on balanced one-way analysis of variance power calculation . Differences of this magnitude represent a minimum threshold that would provide any biological meaning.
Data exclusions	No data were excluded from analyses
Replication	All in vitro experiments were performed using at least 3 biological replicates to ensure reproducibility. For in vivo experiement, each finding was confirmed in a independent and different xenograft model.
Randomization	All mice were randomly assigned into different experimental groups. For in vitro studies, all samples were analyzed equally with no subsampling. Therefore, there was no requirement for randomization.
Blinding	Investigators were generally not blinded as the experimental conditions required investigators to know the identity of the samples.

Reporting for specific materials, systems and methods

We require information from authors about some types of materials, experimental systems and methods used in many studies. Here, indicate whether each material, system or method listed is relevant to your study. If you are not sure if a list item applies to your research, read the appropriate section before selecting a response.

Materials & experimental systems

n/a	Involved in the study
☐	☒ Antibodies
☐	☒ Eukaryotic cell lines
☒	☐ Palaeontology and archaeology
☐	☒ Animals and other organisms
☒	☐ Clinical data
☒	☐ Dual use research of concern

Methods

n/a	Involved in the study
☐	☒ ChIP-seq
☐	☒ Flow cytometry
☒	☐ MRI-based neuroimaging

Antibodies

Antibodies used

Antibody Species Clone Lot. No Supplier Catalog No
anti-RbBP5 Rabbit 3 Bethyl A300-109A
anti-V5 Mouse 212258 Invitrogen 6-0705
Anti-H3K4me3 Rabbit 2871690 EMD/Millipore 17-614
Anti-FOXQ1 Rabbit N/A
anti-ASH2L mouse H3117 SCBT sc-81184
anti-WDR5 Rabbit 1 Bethyl A302-430A
anti-N-Cadherin mouse 32/N-cadherin 9322775 BD 610920
anti-bactin Mouse C4 E0720 SCBT sc-47778
anti-Vimentin Rabbit 8 CST 5741S
anti-Fibronectin Mouse 10/Fibronectin 9070804 BD 610077
anti-Claudin-1 mouse D-4 D1218VL SCBT sc-137121
anti-Occludin mouse E5 JO118 SCBT sc-133256
anti-FLAG mouse SLBT7654 Sigma Aldrich
anti-Myc Rabbit 5 CST 2278S
anti-HA Mouse 2-2.2.14 RJ241582 invitrogen 26183
anti-E-cadherin mouse 36/E-cadherin 1033217 BD 610405
anti-α-catenin mouse 5/a-catenin 31292 BD 610193
anti-b-catenin mouse 14/Beta-catenin 20079 BD 610153
anti-γ-catenin mouse 15/γ-Catenin 15770 BD 610253
Anti-KMT2A/MLL1 Rabbit 5 Bethyl Laboratory A300-374A
Anti-KMT2B/MLL2 Rabbit VL3148318 Invitrogen PA5-103371
Anti-KMT2C/MLL3 Rabbit 129K0565 SIGMA-ALDRICH SAB1300082
Anti-KMT2D/MLL4 Rabbit 3487515 EMD millipore ABE1867
Anti-KMT2E/SET1A Rabbit 7 Bethyl laboratory A300-289A-M
Anti-KMT2F/SET1B Rabbit 1 Bethyl laboratory A302-280A
Horse Anti-Mouse IgG Antibody (H+L), Mouse ZG1208 Vector Laboratories PI-2000-1
anti-Rabbit IgG horse radish peroxidase linked Rabbit 27 CST 7074
PE anti-Human CD24 Mouse ML5 5049759 BD Pharmingen 555428
FITC anti-Human CD44 Mouse G44-26 5275777 Pharmingen 555478
Alexa Fluor 488 goat anti-mouse IgG Mouse 481679 invitrogen A11001
Alexa Fluor 594 goat anti-mouse IgG Mouse 610868 invitrogen A11005

Validation

All Antibodies were validated by the manufacturer. In addition, we validated that all antibodies showed the expected phenotype for a given assay. For almost all antibodies, we validated loss of antibody detection of protein following knockdown ofprotein levels . This was done by either western blot analysis, FACS or confocal microscopy. When we did not validate specificity by knockdown, as was the case for certain antibodies used for western blot analysis, we verified that the antibody yielded the expected

molecular weight and banding pattern.

anti-FOXQ1 We validated it by western blot in different cell models with OXQ1 knockdown and overexpression.
anti-RbBP5 We confirmed that the RbBP5 band at ~75 kDa upon RbBP5 overexpression and knockdown by western blot.
anti-ASH2L We confirmed that the ASH2L band at ~70 kDa upon ASH2L overexpression and knockdown by western blot.
anti-WDR5 We validated that the WDR5 band at ~35 kDa upon WDR5 overexpression and knockdown by western blot.
anti-H3K4me3 We validated this antibody's IP capability by using it in previously used cell lines and qPCR was performed to validate the results are same as previous results for a panel of genes.
anti-bactin We validated a single band at around 45 kDa in different cell lines by western blot
anti-N-Cadherin We observed a single band at the correct molecular weight by western blot
anti-Vimentin We observed a single band at the correct molecular weight by western blot
anti-Fibronectin We observed clean band at the correct molecular weight by western blot
anti-Claudin-1 We observed a single band at the correct molecular weight by western blot
anti-Occludin We observed a single band at the correct molecular weight by western blot
anti-E-cadherin We observed a single band at the correct molecular weight by western blot
anti-α-catenin We observed a single band at the correct molecular weight by western blot
anti-b-catenin We observed a single band at the correct molecular weight by western blot
anti-γ-catenin We observed a single band at the correct molecular weight by western blot
anti-FLAG We validated it by observing correct molecular weight in western blot analysis for several Flag-tagged protein. We also tested Flag Ab by IP proteins tagged with Flag and confirmed in Western blot analysis.
anti-Myc We validated it by observing correct molecular weight in western blot analysis for several Myc-tagged protein. We also tested Myc Ab by IP proteins tagged with Myc and confirmed in Western blot analysis.
anti-HA We validated it by observing correct molecular weight in western blot analysis for several HA-tagged protein. We also tested Ha Ab by IP proteins tagged with Ha and confirmed in Western blot analysis.
anti-V5 We validated it by observing correct molecular weight in western blot analysis for several V5 tagged protein. We also tested V5 Ab by IP proteins tagged with V5 and confirmed it in Western blot analysis.
Anti-KMT2A/MLL1 Rabbit We observed a clean band at the correct molecular weight by western blot
Anti-KMT2B/MLL2 Rabbit We observed a clean band at the correct molecular weight by western blot
Anti-KMT2C/MLL3 Rabbit We observed a clean band at the correct molecular weight by western blot
Anti-KMT2D/MLL4 Rabbit We observed a clean band at the correct molecular weight by western blot
Anti-KMT2E/SET1A Rabbit We observed a cleanband at the correct molecular weight by western blot
Anti-KMT2F/SET1B Rabbit We observed a clean band at the correct molecular weight by western blot

Eukaryotic cell lines

Policy information about cell lines and Sex and Gender in Research

Cell line source(s)

HEK293T Transformed Human kidney cell line ATCC CRL-3216
MDA-MB231 Human breast adenocarcinoma cell line. ATCC CRM-HTB-26
MDA-MB468 Human breast adenocarcinoma cell line. ATCC HTB-132
MDA-MB436 Human breast adenocarcinoma cell line. ATCC HTB-130
SUM1315 Human breast cancer cell line (basal-like), obtainedd from Dr.Stephen P. Ethier.
HMLE Human mammary epithelail cells immortalized with SV40 and hTert, obtained from Dr. Robert A. Weinberg.
HMLER Human mammary epithealil cells transformed by Ras gene in HMLE, obtained from Dr. Robert A. Weinberg.

Authentication

Cells were authenticated by comparing them to the original morphological and growth characteristics and were verified using the GenomeLab short tandem repeat (STR) profiling (Beckman Coulter) with >90% match.

Mycoplasma contamination

All cell lines were tested for mycoplasma negative by DAPI stain and Immunofluorescence microscopy. Only mycoplasma-negative cells were used for research.

Commonly misidentified lines
(See ICLAC register)

No cells from this database were used.

Animals and other research organisms

Policy information about studies involving animals; ARRIVE guidelines recommended for reporting animal research, and Sex and Gender in Research

Laboratory animals

Female NSG mice (8-10 weeks) were purchased from JAX (Jackson Labs).

Wild animals

This study did not involve wild animals

Reporting on sex

This study only used female mice because breast cancer is mainly a female disease.

Field-collected samples

This study did not involve samples collected in the field.

Ethics oversight	All procedures involving mice and experimental protocol (IACUC-19-02-0971) were approved by the institutional Animal Care and Use Committees (IACUC) of Wayne State University.

Note that full information on the approval of the study protocol must also be provided in the manuscript.

ChIP-seq

Data deposition

☒ Confirm that both raw and final processed data have been deposited in a public database such as GEO.

☒ Confirm that you have deposited or provided access to graph files (e.g. BED files) for the called peaks.

Data access links<br>*May remain private before publication.*	GSE141293
Files in database submission	Raw files:<br>102026_ATCACG_S1_H3K4me3_rep1.fastq.gz<br>102027_CGATGT_S2_H3K4me3_rep2.fastq.gz<br>102028_TTAGGC_S3_RbBP5_rep1.fastq.gz<br>102029_TGACCA_S4_RbBP5_rep2.fastq.gz<br>102030_ACAGTG_S5_V5FOXQ1_rep1.fastq.gz<br>102031_GCCAAT_S6_V5FOXQ1_rep2.fastq.gz<br>102032_AGGAAT_S7_inputDNA_rep1.fastq.gz<br>102033_TGCATT_S8_inputDNA_rep2.fastq.gz<br>HMLE-Foxq1-F1_1.fastq.gz<br>HMLE-Foxq1-F1_2.fastq.gz<br>HMLE-Foxq12-F2_1.fastq.gz<br>HMLE-Foxq12-F2_2.fastq.gz<br>HMLE-LacZ1-L1_1.fastq.gz<br>HMLE-LacZ1-L1_2.fastq.gz<br>HMLE-LacZ2-L2_1.fastq.gz<br>HMLE-LacZ2-L2_2.fastq.gz<br>Processed files:<br>rsem_edgeR_FOXQ1.csv<br>FOXQ1_peaks.bed<br>H3K4me3_peaks.bed<br>RbBP5_peaks.bed<br>FOXQ1_RbBP5_DEGtargets.csv<br>FOXQ1_DEDEGtargets.csv
Genome browser session<br>(e.g. UCSC)	http://genome.ucsc.edu/s/avmitch11/FOXQ1_RBBP5

Methodology

Replicates	Experiments were performed in duplicate.
Sequencing depth	ChIP-seq samples were ran on Illumina HiSeq 4000 platform with 50 bp single-end reads. RNA-seq samples were ran on Illumina HiSeq 2000 platform with 100 bp paired-end reads.
Antibodies	anti-RbBP5 (Bethyl, A300-109A), anti-V5(invitrogen, 46-0705), anti-H3K4me3 (ChIPAb+ Trimethyl-Histone H3(Lys4), EMD Millipore, 16-615)
Peak calling parameters	Peak calling was conducted using MACS2 (Galaxy Version 2.1.1.20160309.0) with single-end BAM files as input.<br>We used the following settings:<br>H. sapiens genome (2,451,960,000) was used as reference.<br>Band width of 350 bp<br>Mfold setting: 5-50<br>Minimum FDR (q-value) cutoff for peak detection: 0.001<br>Build model: Shifting model<br>With default parameters:<br>When set, scale the small sample to bigger sample: No<br>Use fixed background lambda as local lambda for every peak region: No<br>When set, use a custom scaling ratio of ChIP/control for linear scaling: 1.0<br>The small nearby region to calculate dynamic lambda: 1000 bp<br>The large nearby region to calculate dynamic lambda: 10000 bp<br>Composite broad regions: No broad regions<br>Use a more sophisticated signal processing approach to find subpeaks summits in each enriched peak region: No<br>How many duplicate tags at the same location are allowed?: 1
Data quality	At FDR 0.1% and 5 Mfold enrichment we identified the following number of peaks that were consistent between sample duplicates for downstream analysis:<br>RbBP5: 25,866

V5-FOXQ1: 13,513
H3K44me3: 18,122

Software

ChIP-seq: The data were analyzed on Galaxy (https://usegalaxy.org/), an open-soure web-based platform. Reads were mapped using Bowtie2 (Version 2.3.2.2) using the built-in Homo sapiens (b37): hg19 reference genome. ChIP-seq peaks were called from alignment results for each biological replicate using MACS2 (Galaxy Version 2.1.1.20160309.0) relative to input, control sample. Peak detection was based on FDR (q-value) set to 0.001. The resulting bedgraph files were converted to bigwis using 'Wig/BedGraph-to-bigWig converter' (Galaxy Version 1.1.1). Enrichment on chromosome and annotation (CEAS) was conducted on peak BED files using Galaxy/ Cistrome (https://cistrome.org/ap) CEAS version 1.0.0. Motif analysis was conducted using peak summits submitted to MEME Suite (Version 5.1.1) at http://meme-suite.org/tools/meme-chip.
RNA-seq: Data was analyzed using R Studio (Version 1.2.5033) and the Bioconductor package (Version 3.1.0). Paired-end reads were mapped to the hg19 human genome using Bowtie2 v2.2.9. The abundance was estimated using RSEM and the differential expression analysis was done using EdgeR v3.12.1 in the Bioconductor package.

Flow Cytometry

Plots

Confirm that:

☒ The axis labels state the marker and fluorochrome used (e.g. CD4-FITC).

☒ The axis scales are clearly visible. Include numbers along axes only for bottom left plot of group (a 'group' is an analysis of identical markers).

☒ All plots are contour plots with outliers or pseudocolor plots.

☒ A numerical value for number of cells or percentage (with statistics) is provided.

Methodology

Sample preparation

Cells were harvested with trypsin and washed with PBS. 2.5 × 10^5 cells were resuspended in 400 microliter PBS. Antibodies against CD44 (FITC, #555478, BD Pharmingen) and CD24 (PE, #555428, BD Pharmingen) were added at 1:200 dilution for 20 mins on ice. Unstained and single stain (CD44 or CD24 alone) samples were generated for compensation and gating controls. Samples were spun down and washed three times with PBS. Just prior to acquisition 10 µL of 1 µg/mL 4′,6-diamidino-2-phenylindole (DAPI) solution was added as a viability dye, detected with a 450/50 bandpass and 406 nm excitation. BD FACS Diva software was used to acquire data, calculate compensation, and export FCS files. BD FACSDiva CS&T Research Beads (BD Biosciences, 655051) were used for instrument QC, and forward scatter area scaling factor was adjusted using cells.

Instrument

Flow cytometry was performed using a BD LSR II (BD Biosciences, San Jose, CA).

Software

BD FACS Diva software was used to acquire data, calculate compensation, and export FCS files.FlowJo software was used for analyzing the results.

Cell population abundance

We did flow analysis without sorting. At least 20,000 cells were collected and analyzed for each analysis.

Gating strategy

Cells were gated using forward scatter area (FCS-A) versus side scatter area (SSC-A) followed by forward scatter width versus s height and side scatter width versus height to select single cells. The viability-dye negative population was selected to exclude dead cells. Populations were then gated as follow markers: CD44 and CD24.

☒ Tick this box to confirm that a figure exemplifying the gating strategy is provided in the Supplementary Information.
