# Supplementary material for: FOXQ1 recruits the MLL complex to activate transcription of EMT and promote breast cancer metastasis
Source: Nat Commun. 2022 Nov 1;13:6548. doi: 10.1038/s41467-022-34239-z (PMC9626503; doi:10.1038/s41467-022-34239-z)

Source data 2 Uncropped blots

Figure 1b

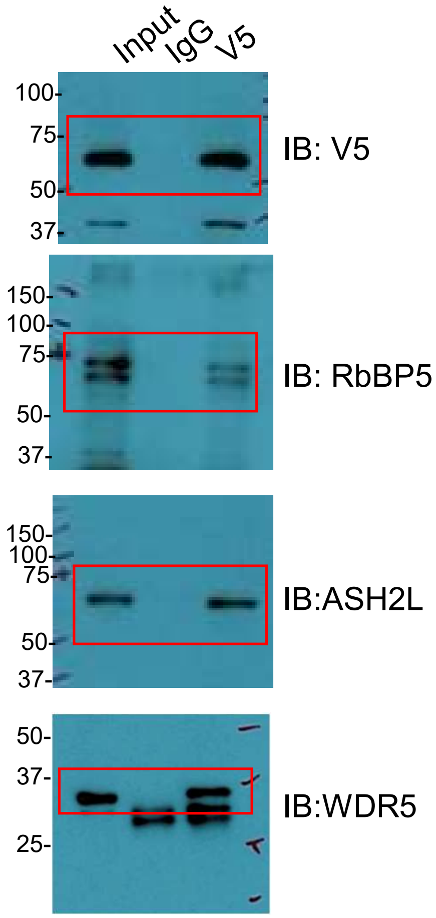

Figure 1c

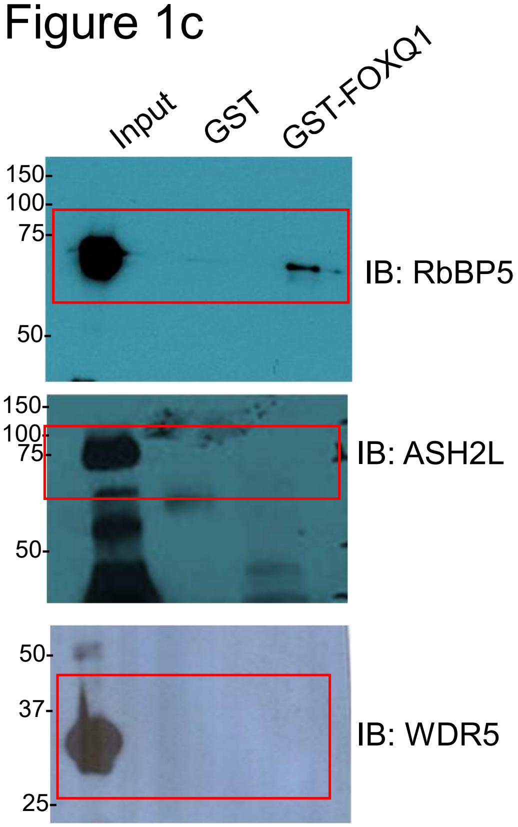

Figure 1d

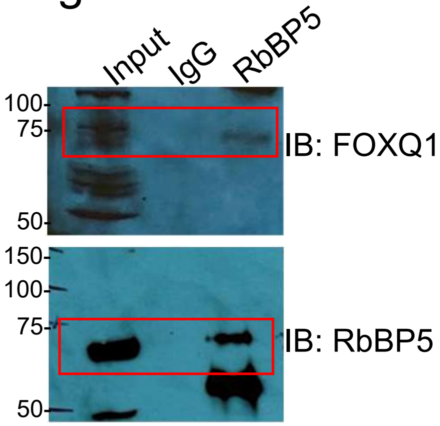

Figure 1e

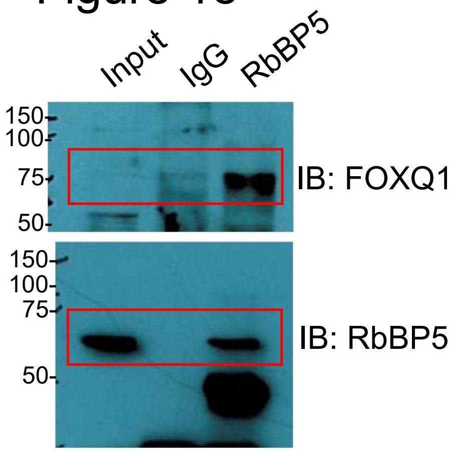

Figure 1f

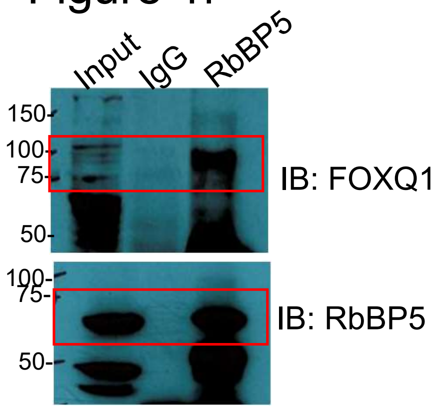

Figure 1g

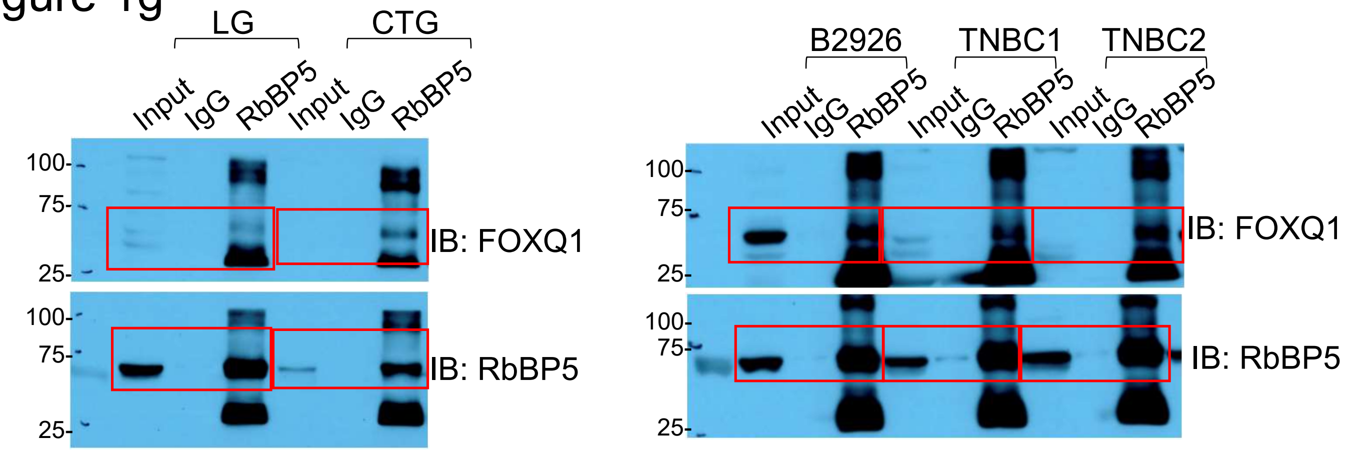

Figure 1h

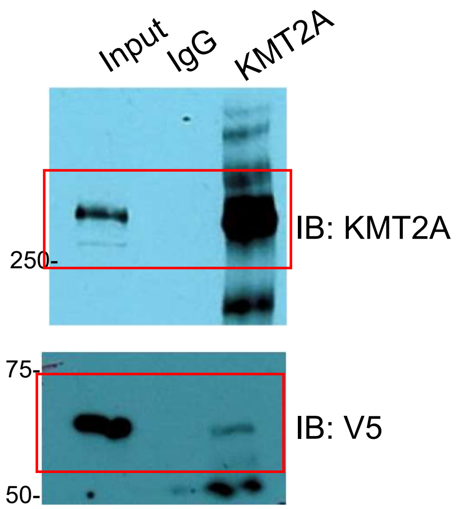

Figure 1i

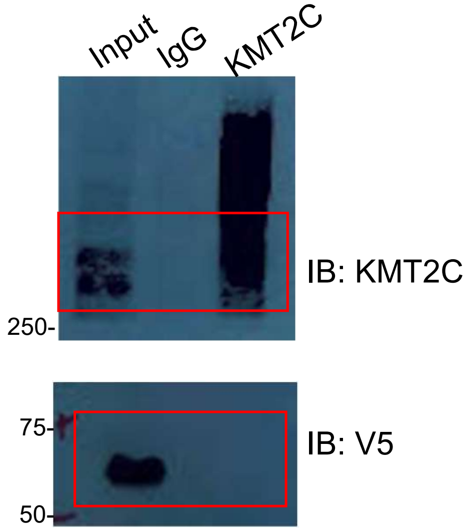

Figure 1j

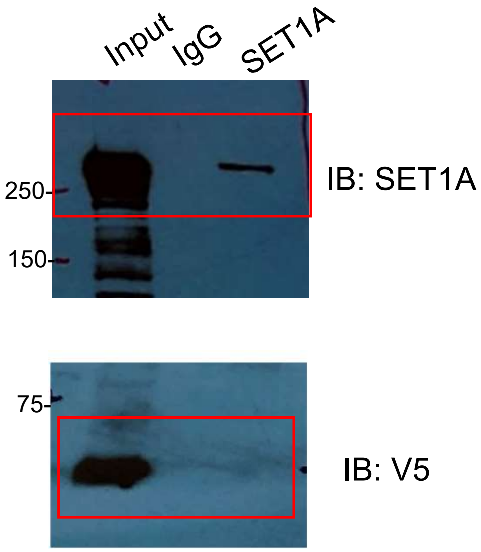

Figure 1k

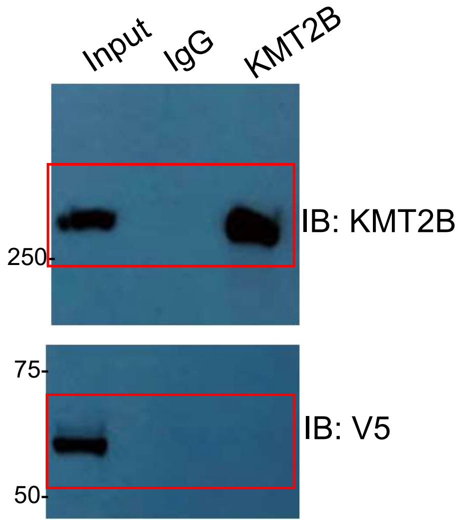

Figure 1l

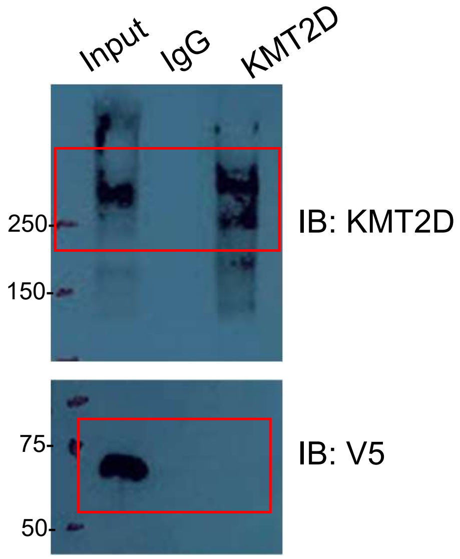

Figure 1m

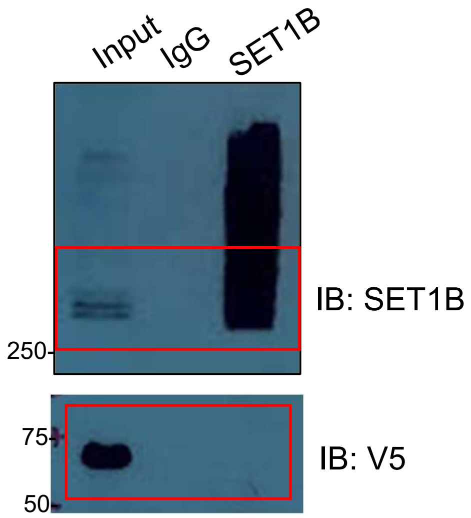

Figure 4b

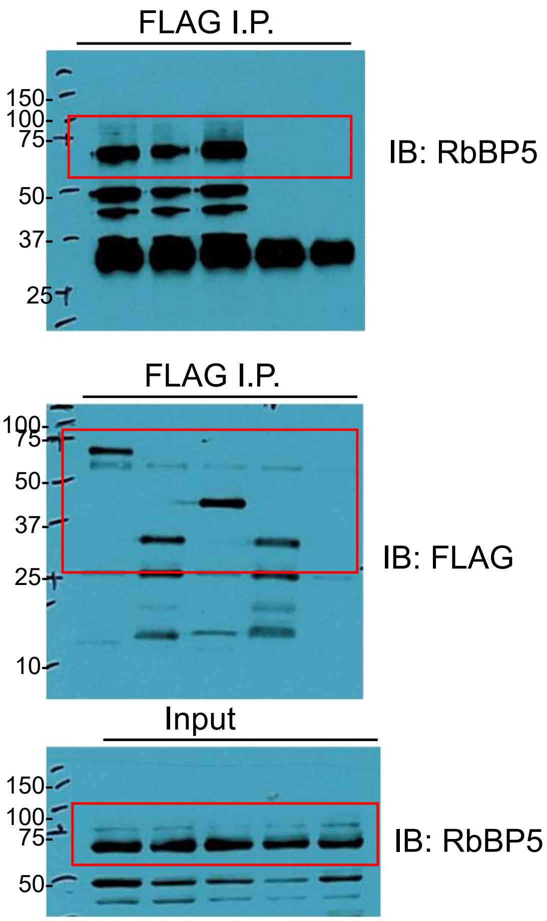

Figure 4d

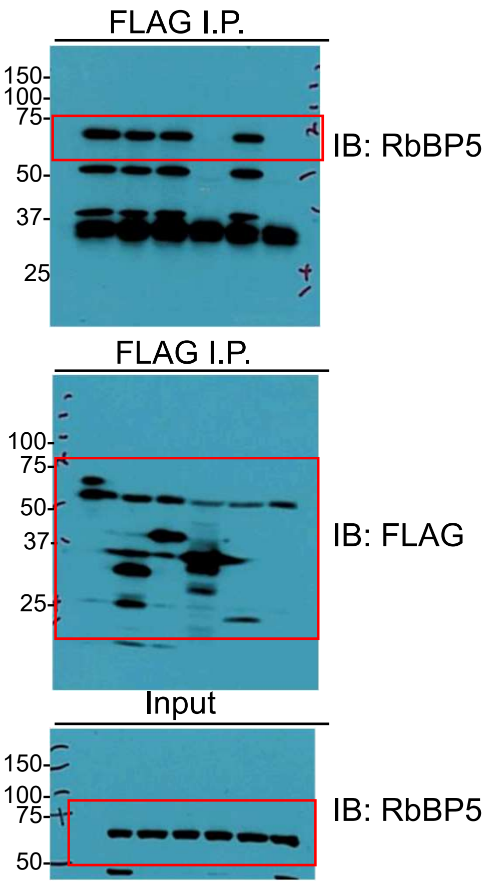

Figure 4f

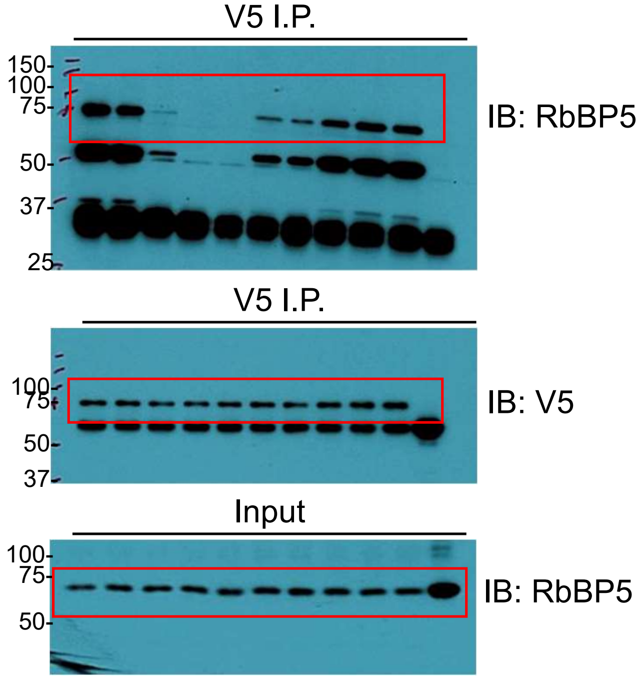

Figure 6c

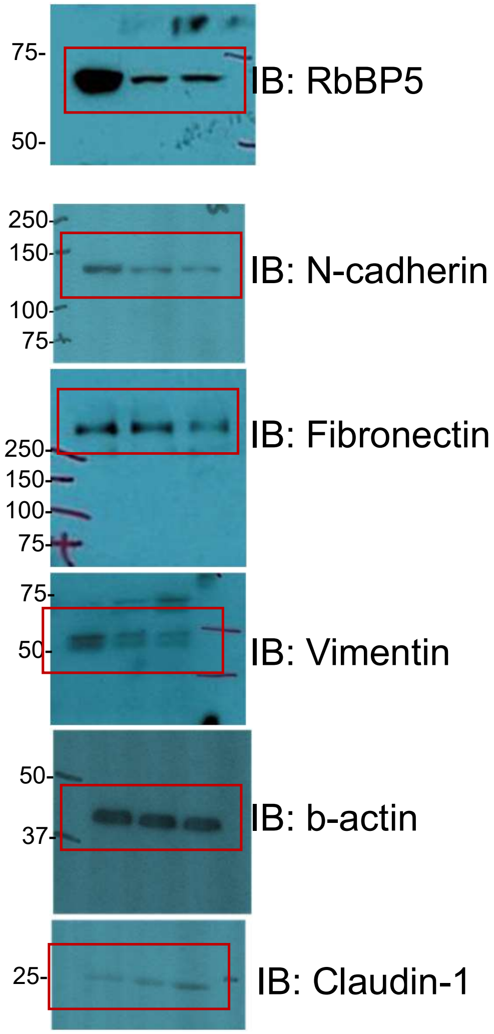

Figure 6h

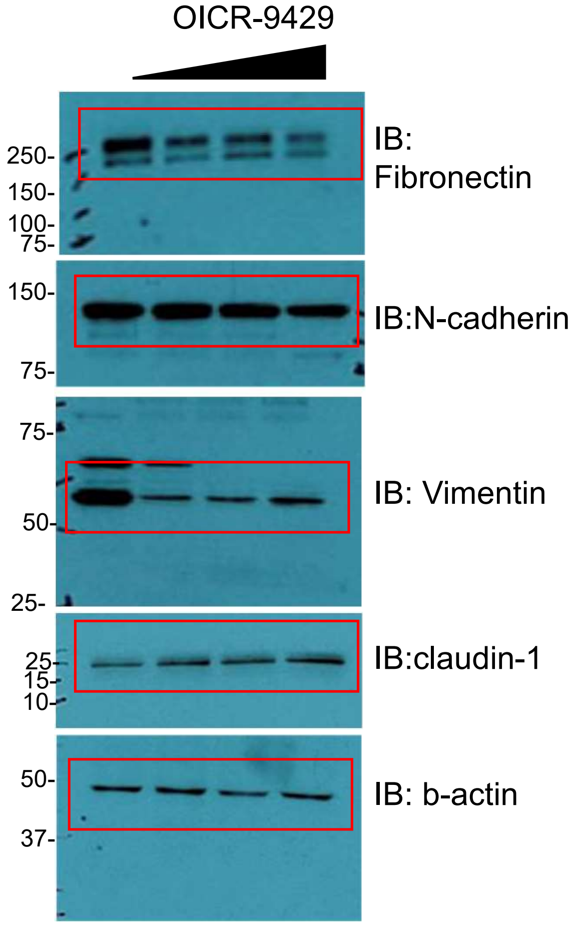

Figure 7a

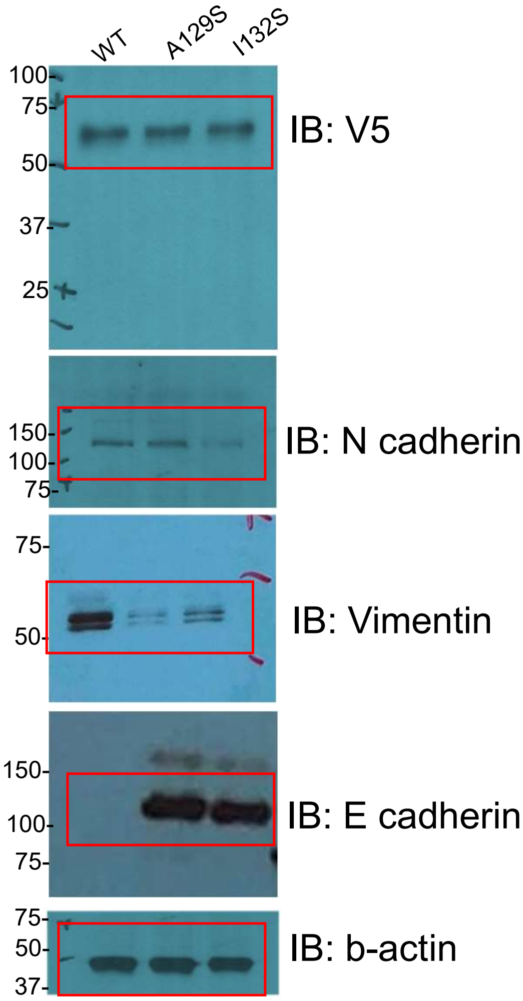

Figure 7f

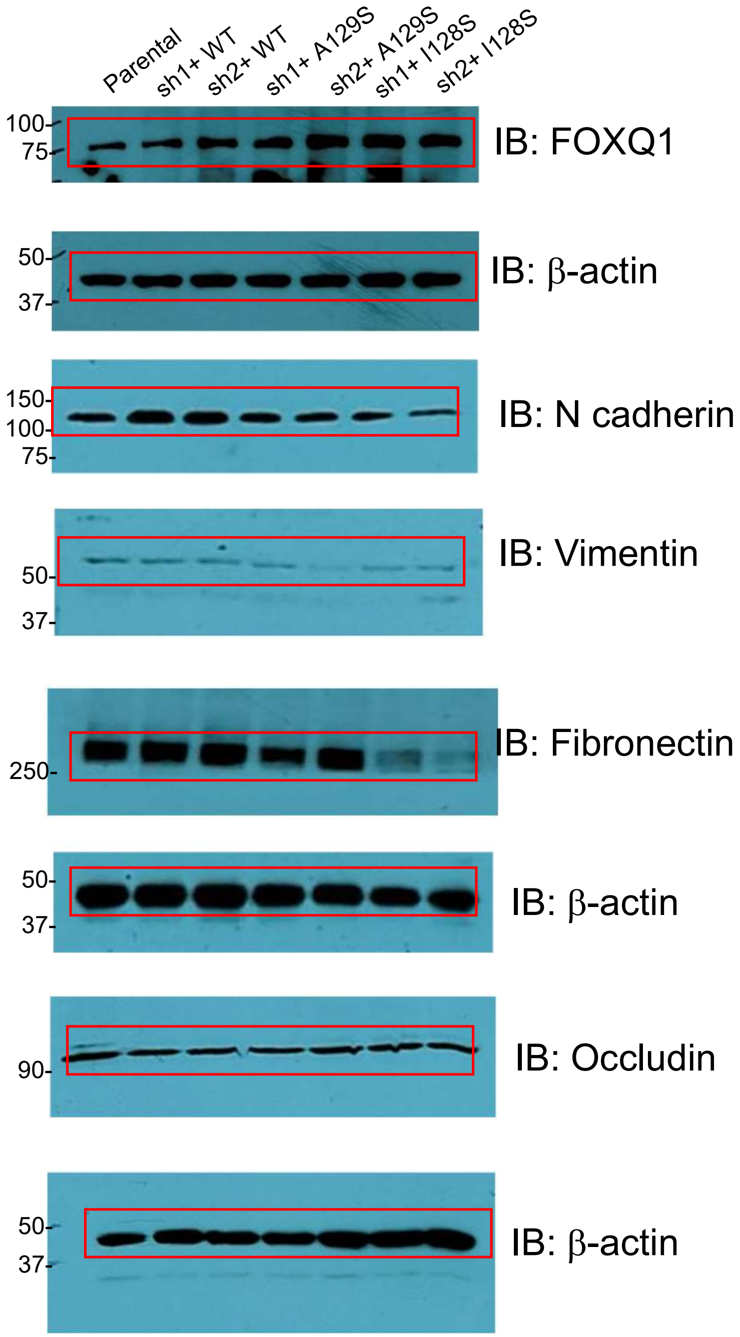

Supplemental figure 1b

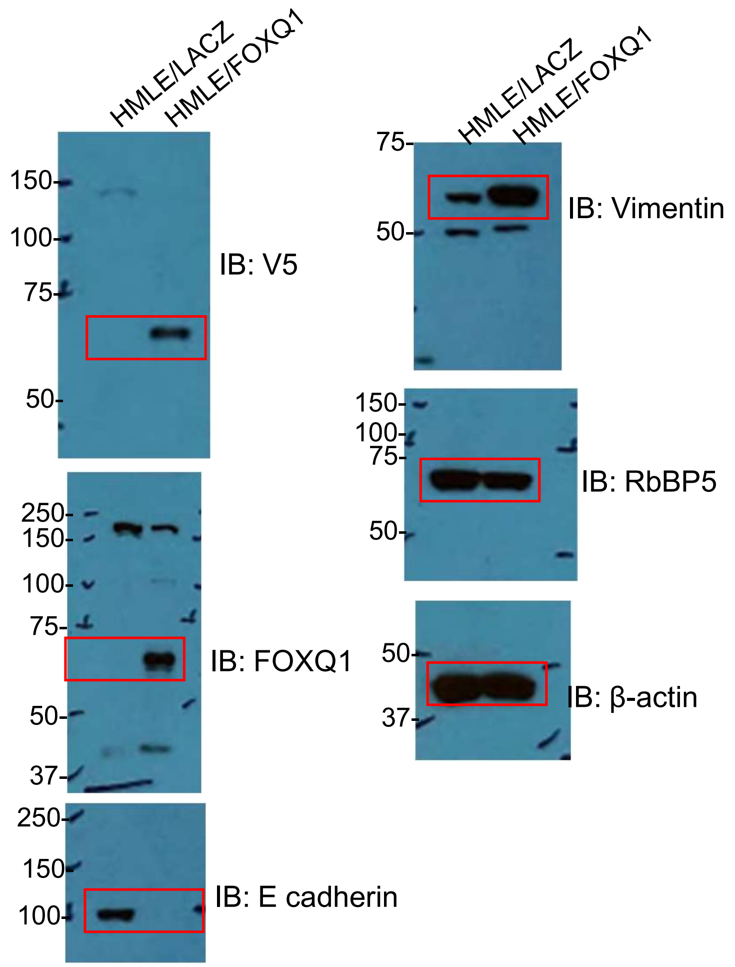

Supplemental figure 1f

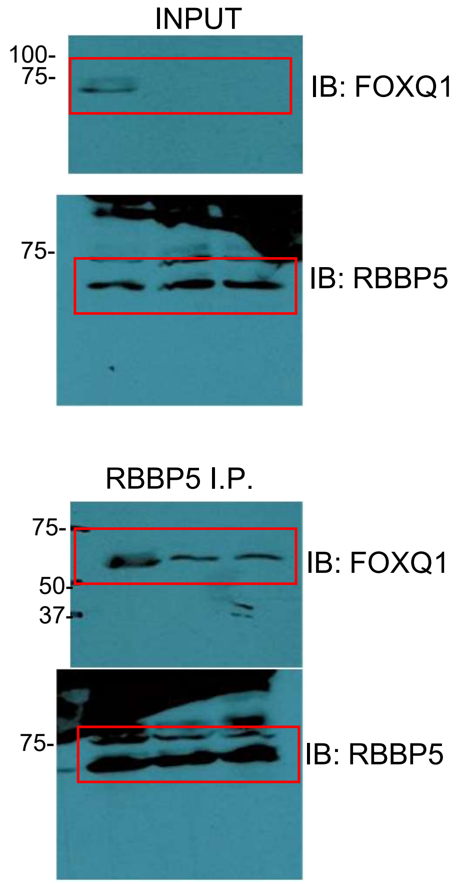

Supplemental figure 1g

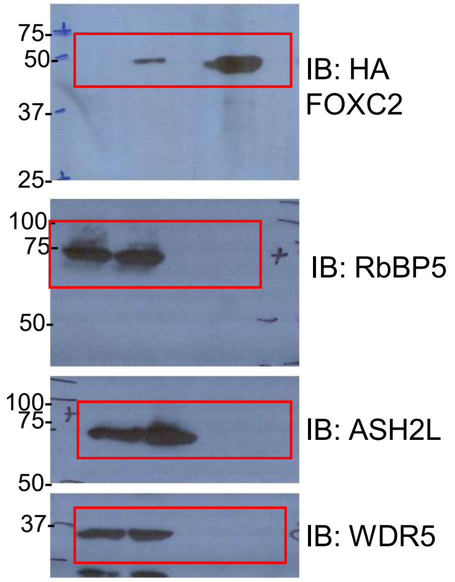

Supplemental figure 1h

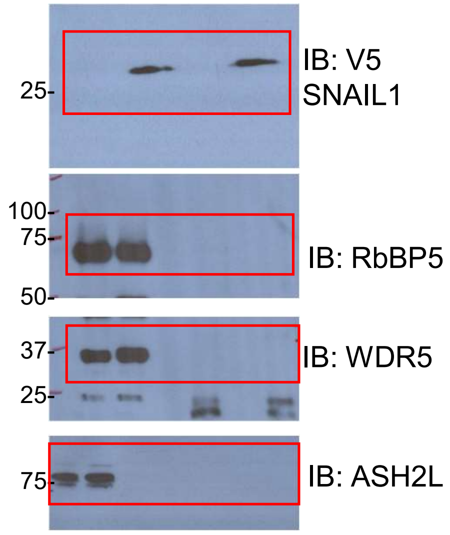

Supplemental figure 3b

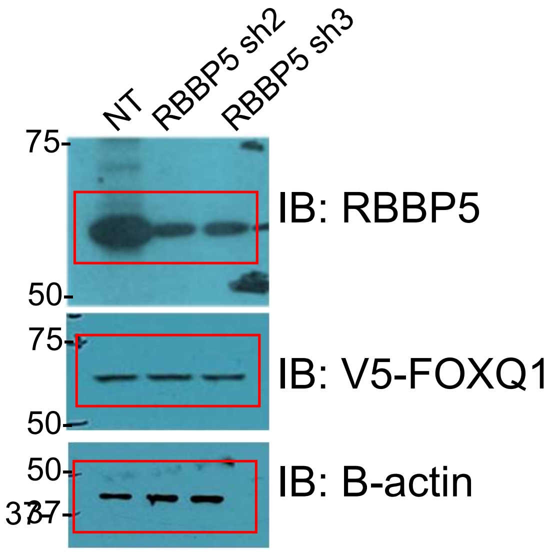

Supplemental figure 3e

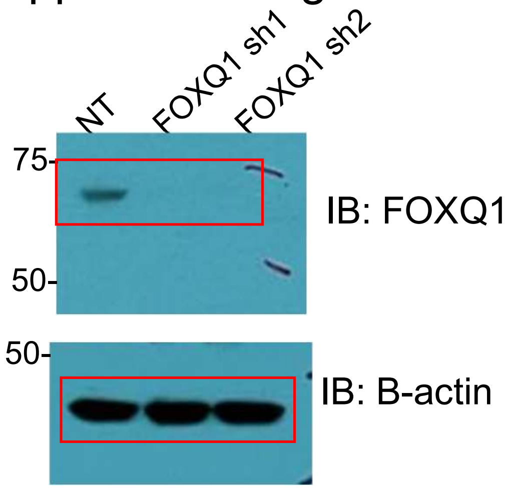

Supplemental figure 3h

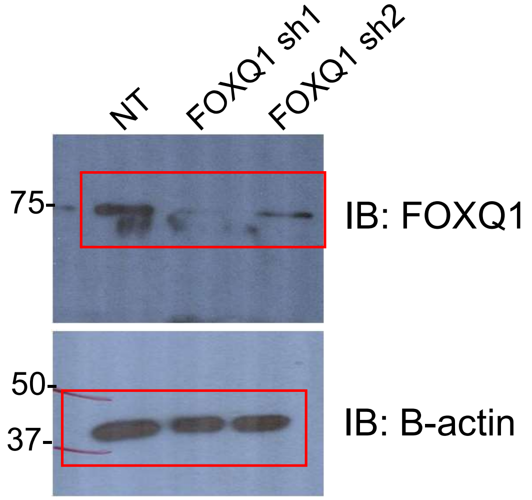

Supplemental figure 3k

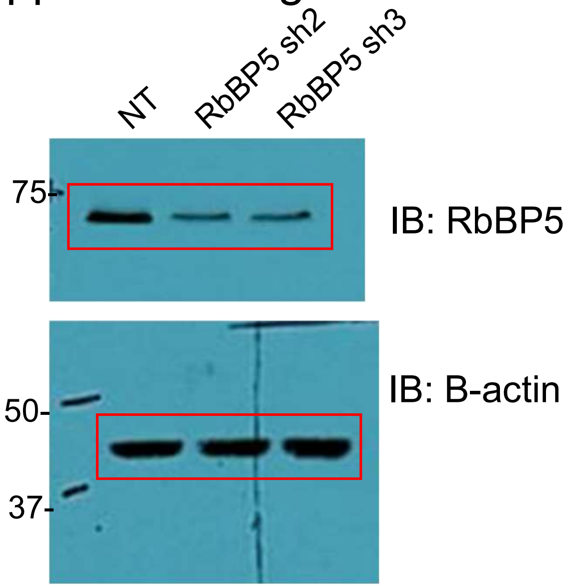

Supplemental figure 3n

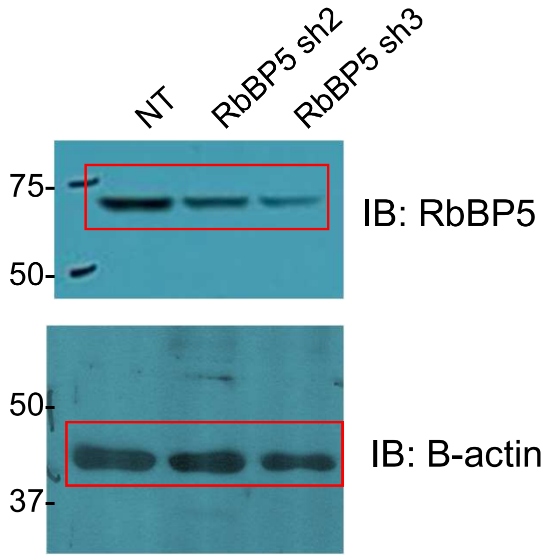

Supplemental figure 3y

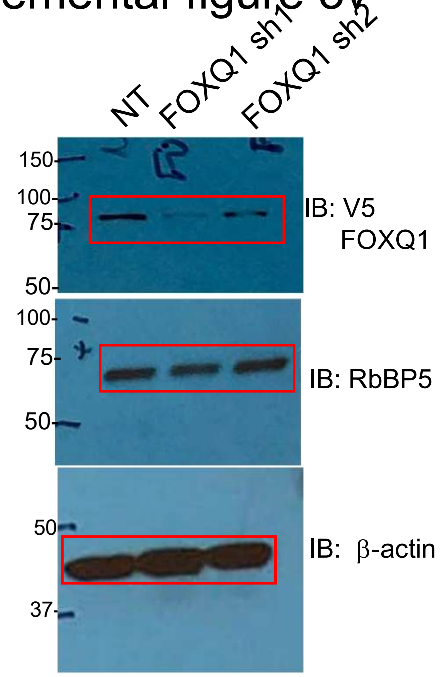

Supplemental figure 4d

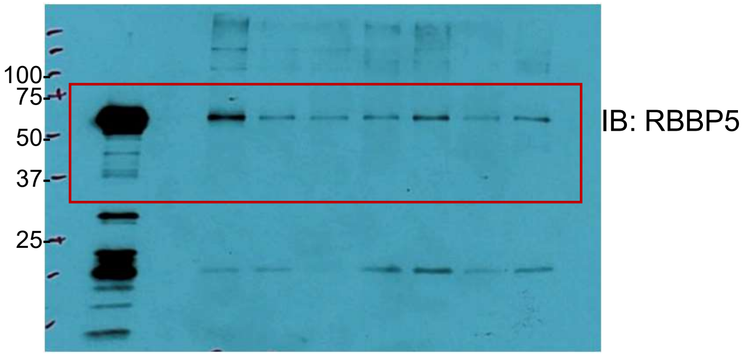

Supplemental figure 4f

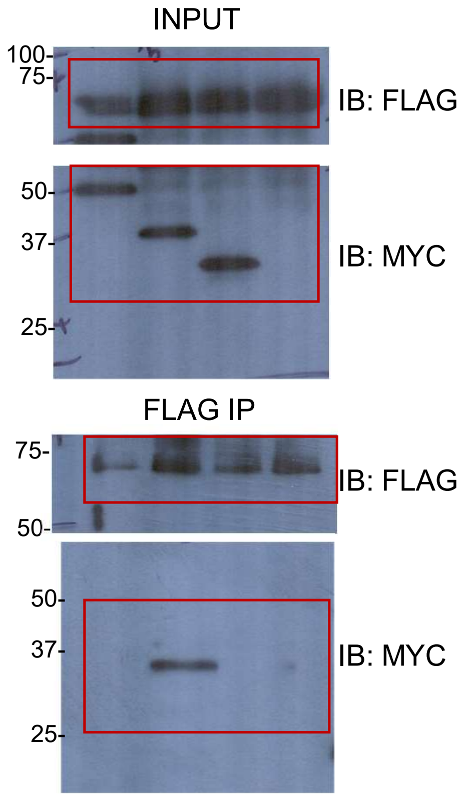

Supplemental Figure 8f

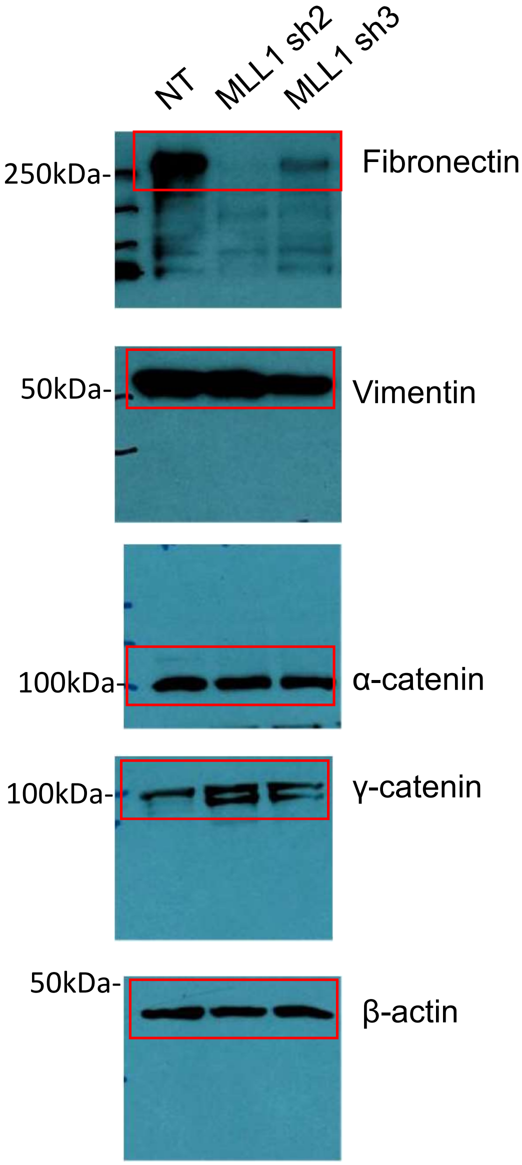

Supplement: Supplementary file 8 — Source Data [file 41467_2022_34239_MOESM8_ESM.zip › Source data 2.pdf]
